# Multiomics Profiling Reveals Signatures of Dysmetabolism in Urban Populations in Central India

**DOI:** 10.3390/microorganisms9071485

**Published:** 2021-07-12

**Authors:** Tanya M. Monaghan, Rima N. Biswas, Rupam R. Nashine, Samidha S. Joshi, Benjamin H. Mullish, Anna M. Seekatz, Jesus Miguens Blanco, Julie A. K. McDonald, Julian R. Marchesi, Tung on Yau, Niki Christodoulou, Maria Hatziapostolou, Maja Pucic-Bakovic, Frano Vuckovic, Filip Klicek, Gordan Lauc, Ning Xue, Tania Dottorini, Shrikant Ambalkar, Ashish Satav, Christos Polytarchou, Animesh Acharjee, Rajpal Singh Kashyap

**Affiliations:** 1 NIHR Nottingham Biomedical Research Centre, University of Nottingham, Nottingham NG7 2UH, UK; 2 Nottingham Digestive Diseases Centre, School of Medicine, University of Nottingham, Nottingham NG7 2UH, UK; 3 Biochemistry Research Laboratory, Dr. G.M. Taori Central India Institute of Medical Sciences, Nagpur 440010, India; rimabiswas13@gmail.com (R.N.B.); rpmnashine@gmail.com (R.R.N.); sjoshi.res@gmail.com (S.S.J.); 4 Division of Digestive Diseases, Department of Metabolism, Digestion and Reproduction, Faculty of Medicine, Imperial College London, London SW7 2AZ, UK; b.mullish@imperial.ac.uk (B.H.M.); j.miguens-blanco18@imperial.ac.uk (J.M.B.); julie.mcdonald@imperial.ac.uk (J.A.K.M.); j.marchesi@imperial.ac.uk (J.R.M.); 5 Department of Biological Sciences, Clemson University, Clemson, SC 29631, USA; aseekatz@clemson.edu; 6 MRC Centre for Molecular Bacteriology and Infection, Imperial College London, London SW7 2AZ, UK; 7 Department of Biosciences, John van Geest Cancer Research Centre, Centre for Health Aging and Understanding Disease, School of Science and Technology, Nottingham Trent University, Nottingham NG7 2UH, UK; payton.yau@ntu.ac.uk (T.o.Y.); niki.christodoulou2019@my.ntu.ac.uk (N.C.); maria.hatziapostolou@ntu.ac.uk (M.H.); 8 Glycoscience Research Laboratory, Genos Ltd., Borongajska cesta 83H, 10000 Zagreb, Croatia; mpucicbakovic@genos.hr (M.P.-B.); fvuckovic@genos.hr (F.V.); fklicek@genos.hr (F.K.); glauc@genos.hr (G.L.); 9 Faculty of Pharmacy and Biochemistry, University of Zagreb, 10000 Zagreb, Croatia; 10 School of Veterinary Medicine and Science, University of Nottingham, Nottingham NG7 2UH, UK; ning.xue@microlise.com (N.X.); tania.Dottorini@nottingham.ac.uk (T.D.); 11 Department of Microbiology and Infection, King’s Mill Hospital, Sherwood Forest Hospitals NHS Trust, Sutton in Ashfield NG17 4JL, UK; shrikant.ambalkar@nhs.net; 12 Mahatma Gandhi Tribal Hospital, MAHAN Trust Melghat, Amravati 605006, India; drashish@mahantrust.org; 13 Institute of Cancer and Genomic Sciences, College of Medical and Dental Sciences, University of Birmingham, Birmingham B15 2TT, UK; 14 Institute of Translational Medicine, University Hospitals Birmingham, Foundation Trust, Birmingham B15 2TT, UK; 15 NIHR Surgical Reconstruction and Microbiology Research Centre, University Hospital Birmingham, Birmingham B15 2WB, UK

**Keywords:** geography, host–microbe interactions, glycome, dysmetabolism, multiomics, diabetes mellitus

## Abstract

Background: Non-communicable diseases (NCDs) have become a major cause of morbidity and mortality in India. Perturbation of host–microbiome interactions may be a key mechanism by which lifestyle-related risk factors such as tobacco use, alcohol consumption, and physical inactivity may influence metabolic health. There is an urgent need to identify relevant dysmetabolic traits for predicting risk of metabolic disorders, such as diabetes, among susceptible Asian Indians where NCDs are a growing epidemic. Methods: Here, we report the first in-depth phenotypic study in which we prospectively enrolled 218 adults from urban and rural areas of Central India and used multiomic profiling to identify relationships between microbial taxa and circulating biomarkers of cardiometabolic risk. Assays included fecal microbiota analysis by 16S ribosomal RNA gene amplicon sequencing, quantification of serum short chain fatty acids by gas chromatography-mass spectrometry, and multiplex assaying of serum diabetic proteins, cytokines, chemokines, and multi-isotype antibodies. Sera was also analysed for *N*-glycans and immunoglobulin G Fc *N*-glycopeptides. Results: Multiple hallmarks of dysmetabolism were identified in urbanites and young overweight adults, the majority of whom did not have a known diagnosis of diabetes. Association analyses revealed several host–microbe and metabolic associations. Conclusions: Host–microbe and metabolic interactions are differentially shaped by body weight and geographic status in Central Indians. Further exploration of these links may help create a molecular-level map for estimating risk of developing metabolic disorders and designing early interventions.

## 1. Introduction

Whilst communicable diseases caused by infectious microbes continue to exert a significant public health burden in India, existing evidence now indicates a marked shift to non-communicable diseases (NCDs) [1,2,3,4]. The consumption of Western-type energy-intense, nutrient poor, high glycaemic index carbohydrate enriched diets, increasingly sedentary occupations, and low levels of recreational activity, particularly in urbanised populations, all lead to a higher body mass index (BMI), evoking a state of chronic metabolic inflammation, termed metainflammation [5,6]. Metainflammation contributes to the development of many NCDs, including diabetes, which has increased rapidly in India over the last quarter of a century, rising from 26 million prevalent cases in 1990 to 65 million in 2016 [7]. The 9th Edition of the IDF Diabetes Atlas in 2019 reported that India is currently home to 77 million diabetics and this number is projected to soar to 134 million cases in the next 25 years. Asian Indians have one of the highest rates of diabetes among major ethnic groups, and the progression from prediabetes to diabetes appears to occur faster in this population [8]. According to the National Urban Diabetes Survey, the estimated prevalence of prediabetes is 14 per cent in India [9]. A more recent study reported that 6 in 10 adults in large South Asian cities have either diabetes or prediabetes [10]. Concerningly, an Indian multistate study has reported that a high percentage of the diabetes cases in the Indian population remain undiagnosed, highlighting issues of poor awareness and detection of diabetes [11]. An important epidemiologic aim going forward will be to identify at-risk individuals, to facilitate an early therapeutic impact.

New multi-biomarker approaches which detect dysmetabolic traits are urgently being sought to predict risk of metabolic diseases such as diabetes and its complications [12]. In this regard, emerging evidence leads us to conclude that metabolic syndrome, which often accompanies obesity and hyperglycaemia, also leads to increased risk of enteric and systemic infections [13]. A recent study suggested that this increased risk may be due to hyperglycaemia, either genetically, chemically or diet-induced, rather than obesity itself, which provides the mechanistic basis for intestinal barrier dysfunction [14].

Alterations in the gut microbiome, metabonome, immune system and, more recently, the total serum and IgG *N-*glycome have been separately described in various dysmetabolic states, with a predominant focus on humans residing in developed nations [15,16,17,18,19,20,21,22,23,24]. However, it remains unclear how these molecular signatures interact, and whether such interactions can offer novel pathophysiological insight into the earliest stages of a dysregulated metabolism that is often associated with an elevated BMI and insulin resistance (IR) state.

To explore this gap in knowledge, we used a multiomics strategy to deeply phenotype rural and urban populations in Central India, unbiasedly sampled in terms of their metabolic state; this unbiased approach allows us to gauge a ‘real world’ cohort without systematically favouring certain populations (e.g., those with metabolic syndrome) over others. We report the first association study investigating the interplay between the circulating immune-metabolic proteome, metabonome, glycome and gut microbiome in previously poorly phenotyped Central Indians. Notably, we associate urban living with multiple hallmarks of metabolic dysregulation, a critical precursor to metabolic disease.

## 2. Materials and Methods

### 2.1. Participant Recruitment

In this observational cohort study, which was carried out during 2019, we prospectively recruited adult (≥18 years of age) participants from both in- and outpatient settings in urban and rural settings of Central India. Health records were also reviewed for each participant where available. Basic demographic details including age, gender, geographic location, as well as information on hospitalisation exposure, antibiotic usage during and before (within 3 months) study recruitment, antacids usage, smoking status, co-morbidities, toilet access, use of hand soap, and presence of domestic animals were recorded for rural and urban participants. Body mass index (BMI) was also recorded for all participants. BMI ranges were pre-defined using WHO Asian BMI classifications: underweight <18.5, normal (18.5–22.9), overweight (23–24.9), pre-obese (25–29.9) and obese (≥30) categories.

Site-specific project coordinators based at the Central Indian Institute of Medical Sciences (CIIMS), Nagpur, and MAHAN Trust, Melghat, supervised recruitment of urban and rural participants and sample acquisition across 25 urban and 35 rural sampling sites, respectively (Table S1). Nagpur is India’s 13th largest city by population (2.5M) and is located at the exact centre of the Indian peninsula. Project fellows approached all consecutive in- and outpatients at CIIMS and also processed samples received from other participating hospitals or private clinical laboratories within a 20 km radius of Nagpur as well as within rural Melghat, Amravati district.

In MAHAN Trust in rural Melghat, which is located approximately 293 km from Nagpur, and is home to a community of 250,000 members of the Korku tribe, all participants were directly recruited by community village healthcare workers and counsellors trained by MAHAN Trust who liaised closely with project fellows from CIIMS. Stool samples were also collected from the rural extensions within a 50 km radius from the satellite centre at MAHAN Trust, Melghat. Here, patient recruitment and sample acquisition were facilitated by village healthcare workers and councillors working in the subdivisional hospitals (SDH) and public health centres. The councillors then contacted the research fellows at the rural satellite hospital in MAHAN Trust.

In contrast to the emerging metropolis of Nagpur, which was declared open defecation free in 2018, and is one of the cleanest and most liveable cities in India, rural agriculturalist communities within Melghat and its rural extension zones are of lower socioeconomic status, display high rates of illiteracy and malnutrition, and possess poor access to medical and educational facilities. Their small hut dwellings are typically composed of mud, grass and bamboo frames which lack an electricity or running water supply or proper sanitation systems. They live in close proximity to their animals (chickens, goats, pigs, cows, buffalo), often in the same one-room dwelling.

### 2.2. Inclusion and Exclusion Criteria

During participant selection, inclusion criteria were (i) adults aged 18 to 70 years of age who could provide written or thumb-print consent, (ii) HIV, hepatitis B or C negative, and (iii) not pregnant or breastfeeding. Participants who were immunosuppressed were not excluded. Immunosuppression was defined as those with cancer, receiving chemotherapy or on prednisolone (>5 mg/d), immunomodulators (azathioprine, methotrexate, calcineurin inhibitor) or biologics. We excluded subjects that were unable to provide a stool sample.

### 2.3. Ethics Statement

This study was approved by the Faculty of Medicine and Health Sciences Research Ethics Committee at the University of Nottingham (REC no. 199-1901) and the Ethical Committee of the Central India Institute of Medical Sciences, Nagpur. All subjects provided verbal and written (or thumbprint) consent.

### 2.4. Sample Preparation

All clinical samples were anonymised and assigned a study code number linked to participant demographic details. Up to two faecal samples (3–5 g each) were collected in UV sterilised dry plastic containers at the time of recruitment from each participant and placed in a cool box. As per the standard operating procedures, all stool specimens were stored at 4 °C immediately after collection to avoid enzymatic degradation prior to genomic DNA extraction which was performed within 24 hours of sample collection. Whole blood samples were drawn from all participants into vacutainer tubes with EDTA as anticoagulant. These were centrifuged for 10 min at 2400× *g* within 30 min of being taken. Serum was then carefully aspirated at room temperature and aliquoted accordingly into single-use cryotubes to avoid repeated freeze–thaw cycles prior to sample storage at −20 °C.

### 2.5. Gut Bacterial Community Profiling by 16S rRNA Gene Sequencing

Stool samples were randomised for processing and DNA was extracted from 1–1.5 g of faeces and homogenised in lysis buffer (Tris HCl, EDTA, NaCl and SDS) using phenol-chloroform method. Briefly, the content was centrifuged at 7000× *g* for 10 min. The supernatant was then transferred to a 1.5 mL tube containing a mixture of isopropanol and sodium acetate (5M) and incubated at −20 °C for 30 min. Following removal of the supernatant the pellet was dried for about an hour. The pellet was suspended in 1X Tris EDTA buffer (pH 8) and incubated at 65 °C for 15 min. An approximate equal volume (0.5–0.7 mL) of phenol: chloroform-isoamyl alcohol (24:1) was added, mixed thoroughly and centrifuged for 10 min at 12,000× *g*. The aqueous viscous supernatant was carefully transferred to a new 1.5 mL tube. An equal volume of chloroform-isoamyl alcohol (1:1) was added, followed by centrifugation for 10 min at 12,000× g. The supernatant was mixed with 0.6×volume of isopropanol to aid precipitation. The precipitated nucleic acids were washed with 75% ethanol, dried and re-suspended in 50μL of TE buffer.

Extracted DNA was quantified using a Qubit 2.0 Fluorometer (ThermoFischer Scientific, Hemel Hempstead, UK), and stored at −80 °C pending downstream assays. Gene-sequencing sample libraries for 16S rRNA were generated via Illumina’s 16S Metagenomic Sequencing Library Preparation Protocol, but with some modifications. Amplification was performed of the V1-V2 16S rRNA gene regions from the faecal DNA, using primers as previously described [25]. Products from the index PCR reactions were cleaned and normalised via the SequalPrep Normalization Plate Kit (Life Technologies, Carlsbad, CA, USA), and library quantification was performed using the NEBNext Library Quant Kit for Illumina (New England Biolabs, Ipswich, MA, USA). Sequencing data were obtained using paired-end 300 bp chemistry on an Illumina MiSeq (Illumina Inc, San Diego, CA, USA), with MiSeq Reagent Kit Volume 3 (Illumina Inc). Sequenced libraries included both negative controls (PCR grade water, Roche, Basel, Switzerland) and positive controls, with the latter using a mock community of 10 bacterial strains (LGC Group, Teddington, UK). Processing of sequencing data was performed via the DADA2 pipeline as previously described [26], using the SILVA bacterial database Volume 132 (https://www.arb-silva.de/ (accessed on 15 February 2021)).

A combination of R packages was used to analyse and visualise microbiota relative abundance data. The inverse Simpson index, non-metric multidimensional scaling (NMDS) and Analysis of Similarities (ANOSIM) were implemented in the R package ‘vegan’ [27], using the Bray–Curtis distance metric based on normalized ASV counts. Partitioning Around Medoids (PAM) clustering [28] on the Jensen–Shannon divergence calculated from normalised ASV counts was used to identify two optimal community types, as defined by best-fit silhouette score (mean silhouette score = 0.47). Linear discriminant analysis Effect Size (LEfSe) [29] as implemented in mothur [30] was used to identify differentially abundant genera in urban vs. rural, or high (≥23) vs. low/normal (<23) BMI score. Kruskal–Wallis and Pearson’s chi-squared tests were run in standard R.

### 2.6. Serum Short Chain Fatty Acid Identification and Quantification

This was performed using a targeted gas chromatography-mass spectrometry protocol, as previously described [31]. Sample analysis was performed on an Agilent 7890B GC system coupled to an Agilent 5977A mass selective detector (Agilent, Santa Clara, CA, USA). Patient samples were run alongside negative controls and quality control samples (pooled aliquots of all patient samples; one run after every ten patient samples) to ensure no source contamination and to assess for signal drift. Three injections were undertaken for each sample. Analysis of data was performed using MassHunter software (Agilent), with SCFA levels calculated via integration of spectra from patient samples and comparison with freshly prepared calibration curves using SCFA standards (Merck, Darmstadt, Germany).

### 2.7. Serum N-Glycome Profiling

#### 2.7.1. Experimental Design

Participant serum samples and in-house serum standards were thawed, vortexed and centrifuged for 3 min at 12,100 *g*. Each sample (100 μL) was aliquoted to 2 mL 96-well collection plates (Waters, Milford, MA, USA) following a predetermined, established experimental design [32] which included blocking of all known sources of variation (age, sex, diarrheal/non-diarrheal and urban/rural status) and sample randomization between the plates to reduce experimental error. In-house serum standards were aliquoted in seven to eight replicates per plate, to evaluate experimental error and integrity of generated data. An aliquot (10 μL) of each sample was transferred to 1 mL 96-well collection plates (Waters, Milford, MA, USA) for *N*-glycome analysis and the rest was used for isolation of IgG followed by IgG Fc *N*-glycopeptide analysis.

#### 2.7.2. Serum N-Glycome Analysis

Serum *N*-glycans were enzymatically released from proteins by PNGase F, fluorescently labelled with 2-aminobenzamide and cleaned-up from the excess of reagents by hydrophilic interaction liquid chromatography-solid phase extraction (HILIC-SPE), as previously described [33]. Fluorescently labelled and purified *N*-glycans were separated by HILIC on a Waters BEH Glycan chromatography column, 150 × 2.1 mm i.d., 1.7 μm BEH particles, installed on an Acquity ultra-performance liquid chromatography (UPLC) H-class system (Waters, Wilmslow, UK), consisting of a quaternary solvent manager, sample manager and a fluorescence detector set with excitation and emission wavelengths of 250 nm and 428 nm, respectively. Obtained chromatograms were separated into 39 peaks. The amount of *N*-glycans in each chromatographic peak was expressed as a percentage of total integrated area. From 39 directly measured glycan peaks we calculated 12 derived traits which average particular glycosylation traits such as galactosylation, sialylation and branching across different individual glycan structures and are, consequently, more closely related to individual enzymatic activities and underlying genetic polymorphisms. Derived traits used: the proportion of low branching (LB); defined as di-antennary complex type *N*-glycans with two *N*-acetylglucosamine residues attached to the core pentasaccharide, (Man3GlcNAc2) at both the α-3 and α-6 mannose sites and high branching (HB); tri- and tetra-antennary complex type *N*-glycans with three of four *N*-acetylglucosamine (GlcNAc) residues attached to the core pentasaccharide. The majority of antennas are further elongated by the addition of galactose, sialic acid and fucose. Additional modifications such as the addition of bisecting GlcNAc and/or a fucose residue on the core pentasaccharide are also possible. *N*-glycans, the proportion of a-, mono-, di-, tri- and tetra-galactosylated *N*-glycans (G0, G1, G2, G3 and G4, respectively), and a-, mono-, di-, tri- and tetra-sialylated *N*-glycans (S0, S1, S2, S3 and S4, respectively).

#### 2.7.3. IgG Fc N-Glycopeptides Analysis

Sample preparation and analysis of IgG *N*-glycopeptides was done following a previously described protocol with minor changes [34]. Briefly, IgG was isolated from 90 µL of serum samples by affinity chromatography using CIM^®^ 96-well Protein G monolithic plate (BIA Separations, Ajdovščina, Slovenia). IgG *N*-glycopeptides were prepared by trypsin digestion of an aliquot of IgG isolates (25 μg on average per sample) followed by reverse-phase solid phase extraction (RP-SPE). Purified tryptic IgG *N*-glycopeptides were separated and measured on nanoAcquity chromatographic system (Waters, Wilmslow, UK) coupled to Compact Q-TOF mass spectrometer (Bruker, Bremen, Germany), equipped with Apollo II source and operated under HyStar software version 3.2. The first four isotopic peaks of doubly and triply charged signals, belonging to the same glycopeptide species, were summed together, resulting in 20 Fc *N*-glycopeptides per IgG subclass. Predominant allotype variant of IgG3 tryptic peptide carrying *N*-glycans in the Caucasian population has the same amino acid sequence as IgG2 [35]. Therefore, IgG glycopeptides were separated into three chromatographic peaks designated as IgG1, IgG2/3 and IgG4. Signals of interest were normalised to the total area of each IgG subclass.

### 2.8. Immune and Diabetic Protein Profiling of Sera

Patient sera were analysed for the quantifications of 37 key biomarkers of inflammation from the TNF superfamily proteins, IFN family proteins, Treg cytokines, and MMPs: APRIL/TNFSF13, BAFF/TNFSF13B, sCD30/TNFRSF8, sCD163, Chitinase-3-like 1, gp130/sIL-6Rβ, IFN-α2, IFN-β, IFN-γ, IL-2, sIL-6Rα, IL-8, IL-10, IL-11, IL-12 (p40), IL-12 (p70), IL-19, IL-20, IL-22, IL-26, IL-27 (p28), IL-28A/IFN-λ2, IL-29/IFN-λ1, IL-32, IL-34, IL-35, LIGHT/TNFSF14, MMP-1, MMP-2, MMP-3, Osteocalcin, Osteopontin, Pentraxin-3, sTNF-R1, sTNF-R2, TSLP, TWEAK/TNFSF12 using the Bio-Plex Pro Human Inflammation Panel 1 (171AL001M, Bio-Rad, Hercules, CA, USA); immunoglobulins IgG1, IgG2, IgG3, IgG4, lgA, lgM, using the Bio-Plex Pro™ Human Isotyping Panel (171A3100M, Bio-Rad); and C-peptide, ghrelin, GIP, GLP-1, glucagon, insulin, leptin, PAI-1 (total), resistin and visfatin, using the Bio-Plex 10 Pro^TM^ Human diabetes 10-plex immunoassay (171A7001M, Bio-Rad), respectively. Samples were analysed in a Bio-Plex 200 System using the Bio-Plex manager software, according to manufacturer’s instructions. The concentrations were calculated by standard curves developed in parallel and are expressed as pg/mL for the inflammatory biomarkers and diabetic proteins, and ng/mL for the immunoglobulins.

Glycated serum protein (GSP) levels (μmol/L), which provide a short to medium-term assessment of glycaemia and diabetes risk [36], were assayed in sera by enzymatic assay (Crystal Chem, Elk Grove Village, IL, USA).

### 2.9. Statistical Analysis 

As per the manufacturer’s guidelines, all sera were assayed in duplicate in immune (antibody and inflammation panels), diabetic protein, and GSP assays. Descriptive statistics including median and interquartile range (IQR) are presented for demographic variables. Student’s t-tests were used to detect differences in the abundance of microbial and immunometabolic features across the groups assessed. The association between the metavariables and microbial taxa was assessed using Pearson’s correlation analysis. Identification and selection of the candidate biomarkers associated with urban, rural and BMI status, together with the performance of markers, was investigated using the elastic net method (see below) [37]. All *p*-values were adjusted where necessary to control for the false discovery rate (FDR) according to the Benjamini–Hochberg method. All analyses were performed in the R statistical computing (R version 3.4.3) environment. Statistical significance was set at an alpha of 5% for a two-sided *p*-value for all analyses.

#### Elastic Net Machine Learning Method

We the applied elastic net (EN) machine learning method [37] to help select important features which may discriminate between the urban and rural population, and BMI groups. Elastic net automatically selects the best features linked with the outcome or response variable from the dataset-based penalty applied, and hence provides a sparse solution [38,39,40]. Penalty parameters, λ (Range of λ: 0 to 1), are optimized using 10-fold cross validation. The stronger the penalty (close to 1), the smaller the number of variables selected, while if the penalty is weaker (close to 0), a higher number of variables are selected. In other words, the penalty function λ controls the trade-off between likelihood and penalty, thereby influencing the variables to be selected. Elastic net employs a mixed version of penalty called L1 (Least Absolute Shrinkage and Selection Operator also called as LASSO penalty) and L2 penalty (Ridge penalty). The L1 penalty encourages the sparse representation, whereas L2 stabilises the solution. The process was repeated 100 times and the features were ranked according to their respective selection frequency associated with each run. We then selected the first quartile from the EN-selected features over 100 runs. These selected features were then further modeled by generating area under curve (AUC) curves. We performed stability analysis [39] (also called a permutation analysis) after randomizing the class label (for rural vs. urban populations). We compared a random AUC based on each iteration and averaged over 100 iterations with the true AUC (without changing the class label). From these calculations, we generated two AUC distributions and compared mean values of the distributions, and generated *p*-values accordingly. All analyses were performed in the R statistical computing (R version 3.4.3) environment [41] and MetaboAnalyst web tool [42].

## 3. Results

### 3.1. Characteristics of the Study Participants

Clinical and demographic characteristics of the cohort are presented in Table 1 and geographic sites specified in Table S1. In total, 218 participants, of which 26.6% were inpatients, were enrolled into this prospective cohort study during 2019. A survey of the medical records of the urban cohort revealed that 10.5% of participants had diabetes mellitus at enrolment. No co-morbidity data were available for the rural cohort, highlighting a lack of diagnostic hospital facilities, a general reluctance to engage with Western medicine and a reliance on local faith healers and alternative medicines. In terms of cardiometabolic risk factors, 24.5% were active smokers (rural *n* = 23 vs. urban *n* = 31), and over half of the cohort were overweight (BMI ≥ 23) by Asian WHO standards. The urban Nagpurian cohort displayed significantly higher BMIs compared to their rural counterparts (*p* < 0.001).

**Figure 1:**
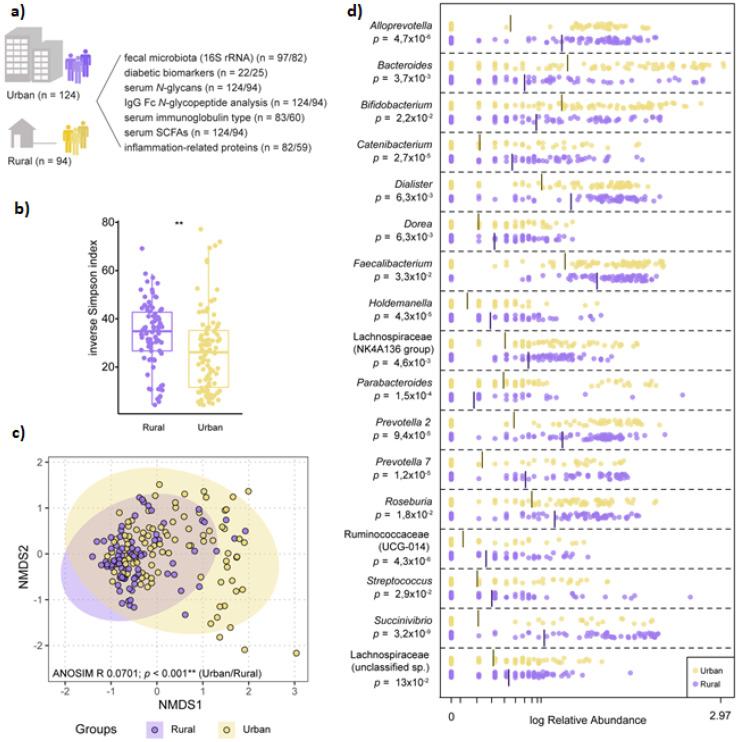
The microbiota is structurally distinct in participants from rural vs. urban areas. (**a**) Schematic of overall study design (*n* = number of urban/rural samples). (**b**) Diversity as determined by inverse Simpson index based on normalized ASV counts in participants from rural vs. urban areas (Kruskall–Wallis nonparametric test, *p* < 0.001). (**c**) Non-metric multidimensional scaling (NMDS) visualization of Bray–Curtis distance (based on normalized ASV counts) of the microbiota in participants based on geography (rural vs. urban; purple vs. yellow). Analysis of similarities (ANOSIM) was conducted using Bray–Curtis distance, 9999 permutations. (**d**) Log-transformed relative abundance of significantly differential genera between participants from rural or urban areas, as determined by Linear discriminant analysis Effect Size (LEfSe).

Following quality control checks, we analysed 179 fecal samples for taxonomic composition by 16S rRNA gene amplicon sequencing. Sera were profiled for relative abundance of total serum *N*-glycans and IgG Fc *N*-glycopeptides (*n* = 218), detection and quantification of short chain fatty acids (*n* = 218), an inflammation panel of immune proteins (*n* = 141), a multi-isotype antibody panel (*n* = 143), glycated serum protein levels (*n* = 135), and a diabetes panel (*n* = 47); see Figure 1A for study schematic with urban/rural sampling numbers and Supplementary Table S2 for study metrics.

### 3.2. Microbiota Composition Varies by Geographic-Specific Factors

Significant differences in microbiota diversity, structure, and composition were observed between urban and rural participants. Overall, microbiota diversity was increased in the rural population (Figure 1B), and ANOSIM on NMDS ordination indicated significant separation between the two groups (Figure 1C). LEfSe identified several overrepresented genera belonging to the Firmicutes phylum in the rural population, including significant differences in relative abundance of *Faecalibacterium, Roseburia*, *unclassified Lachnospiraceae* and *Ruminococcaceae* groups. Within Bacteroidetes, the rural microbiota was dominated by *Prevotella* and *Alloprevotella* genera, while *Bacteroides* and *Parabacteroides* were overrepresented in the urban microbiota (Figure 1D). Community type analysis using PAM clustering revealed two major clusters, with an overrepresentation of rural samples clustering within one cluster (69/82) compared to urban samples, which were more evenly distributed between both clusters (56 vs. 41 samples; Pearson’s chi-squared test, *p* < 0.001).

BMI (defined as ‘low/normal’ <18.5/18.5–22.9 vs. ‘high’ >23) was not a significant factor in differentiating microbiota composition or diversity; however, an unclassified *Ruminococcaceae* group (*Ruminococcaceae_UCG-014*) was overrepresented in participants with a high BMI across all samples and within rural participants (online Supplementary Figure S1).

### 3.3. Dysmetabolic Hallmarks and Urban Living

Diabetic biomarker panel profiling revealed substantially higher levels of proteins linked to diabetes including C-peptide, insulin and leptin in the peripheral circulation of the sampled urban population (Table 2). Accompanying serum *N*-glycan profiles demonstrated glycan structural features of increased branching, galactosylation and sialylation in the urban cohort, in line with increasing plasma *N*-glycome complexity typically observed in individuals with increased risk of type 2 diabetes development [17]. Specifically, we found a statistically significant increase in levels of high-branching, tri-and tetragalactosylated glycans, tri-and tetrasialylated glycans and increase in levels of glycans with antennary fucosylation in inhabitants of Nagpur. For IgG Fc *N*-glycopeptide analysis, IgG1 glycopeptides with agalactosylated and monogalactosylated glycans were detected at a significantly higher relative abundance in the sera of tested urban inhabitants.

### 3.4. Rural Living Associates with Contrasting Serum Immunometabolic Features

Levels of a number of short chain fatty acids (including caproate, valerate, acetate and propionate) were significantly higher in the sera of rural inhabitants. Rural inhabitants showed a significantly higher relative abundance of low branching, monosialylated and digalactosylated serum glycans, as well as a higher abundance of bisected and high mannose serum glycans (Table 2). Analysis of IgG Fc *N*-glycopeptides revealed a higher relative abundance of IgG1 and IgG4 glycopeptides with digalactosylated and monosialylated glycans with core fucose in the circulation of rural subjects.

**Figure 2:**
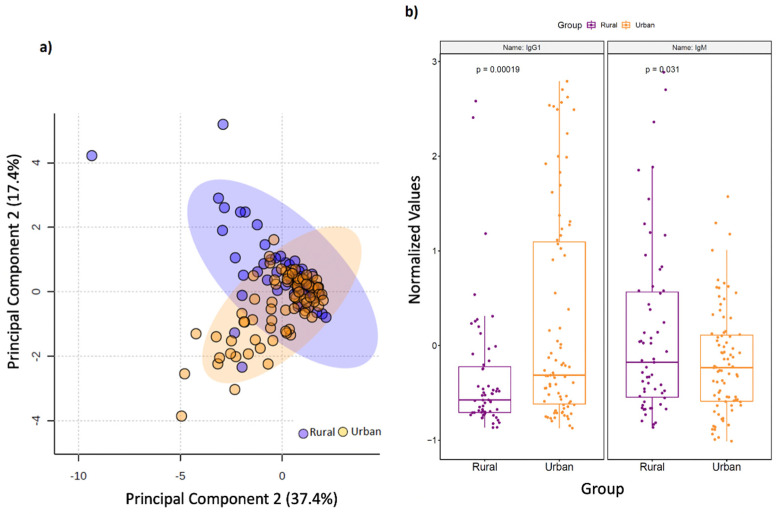
Serum immunoglobulin levels vary by geography. (**a**) Principal component analysis (PCA) score plot on the selected features demonstrates a clear separation in serum multi-isotype antibody responses in terms of geographic setting of sampled population. Dots represent patients and are coloured according to the subject cohort. Ellipse represents 95% confidence. Results are plotted according to the Principal component-1 (PC1) and Principal component-2 (PC2) scores, with the percent variation of the cohort explained by the respective x and y axess. (**b**) Box plots showing levels of serum IgM and IgG1 antibodies in rural and urban cohorts, respectively.

Geographic differences also extended to circulating immunoglobulin responses. Principal component analysis (PCA) (Figure 2A) demonstrated a clear separation of multi-isotype antibody responses between rural and urban cohorts. In particular, the rural cohort displayed significantly higher levels of circulating total IgM antibodies, whereas IgG1 antibodies were significantly higher in the urban cohort (*p* < 0.05; Figure 2B). Correlation analyses also focussed on studying connections between immunoglobulin responses and SCFAs, the latter of which are known to fuel antibody responses. Here, we found that serum 2-hydroxybutyrate positively correlated with IgG4 levels in the rural cohort (*p* < 0.05), and IgG4 strongly positively associated with *Porphyromonas*, *Campylobacte*r, *Gemella*, *Streptoccocus*, *Leptotrichia* and *Neisseria* (*p* = 0).

### 3.5. Diabetic Protein-Microbe Interactions Vary by Geography

In the urban group (see online Supplementary Table S3), the strongest positive Pearson correlations were detected between visfatin and *Bacillales*, *Marinifilaceae, Staphylococcaeae, Odoribacter*, *Macelibacteroides*, *Staphylococcus*, *Hungatella*, *Ruminiclostridium_6*, *Erysipelatoclostridium*, *Acidaminococcus*, and *Lactobacillaceae* (*p* < 0.0001); followed by leptin with *Actinobacteria*, and *Bifidobacteriales* at class, family and genus. Positive correlations were observed for GLP-1 with *Escherichia/Shigella*, *Enterobacteriaceae*, *Proteobacteria* and *Gammaproteobacteria*. C-peptide and insulin also positively associated with *Gammaproteobacteria*, *Proteobacteria* and *Enterobactericeae*.

In contrast, in the rural group, (see online Supplementary Table S4), the strongest positive correlations were detected for GLP-1 with unclassified *Erysipelotrichaceae_unclassfied*, *Anaeroplasmatales* at class, family and genus, and *Erysipelotrichaceae_UCG.004*, and for C-peptide, with *Paraprevotella*, *Flavonifractor*, *UBA1819* and *Erysipelatoclostridium* (*p* < 0.0001). Further significant diabetic protein–microbiota–immune correlations for the urban and rural groups are presented in online Supplementary Tables S3 and S4, respectively.

### 3.6. Differential Impact of Glycated Serum Protein Levels on Immunometabolic and Gut Bacterial Features

In subjects where glycated serum protein (GSP) concentrations were assessed (*n* = 135), levels were significantly higher in overweight (BMI 23–24.9) and pre-obese (BM 25–29.9) test subjects compared to those with normal BMI (18.5–22.9), and in urban subjects compared to rural participants; *p* < 0.001 (Table S5). Across the whole cohort, high GSP levels were associated with significantly lower circulating IgG2, IgM, caproate, and valerate levels, and lower relative abundance of *Roseburia* and *Dorea* (*p* < 0.05). See Table 3, Table 4 and Table 5.

In urban BMI comparisons, circulating levels of 2-hydroxybutyrate, isobutyrate, propionate, and valerate were significantly higher in overweight subjects compared to those with normal BMI (see Supplementary Table S6; *p* < 0.05). Similarly, levels of isobutyrate, propionate and valerate, alongside acetate were higher in pre-obese vs. normal BMI subjects (see online Supplementary Table S7). Contrastingly, in rural-BMI group comparisons, pre-obese subjects displayed significantly lower levels of 2-methylbutyrate, acetate, caproate, isobutyrate and isovalerate, but a higher relative abundance of *Collinsella, Prevotella_9, Agathobacter, Roseburia, Faecalibacterium, Ruminococcaceae unclassified, Ruminococcaceae_UCG.014, Catenibacterium*, *Megasphaera* and *Mitsuokella* compared to subjects with a normal BMI (*p* < 0.05; see online Supplementary Table S8). In underweight rural subjects (*n* = 8), there was a higher representation of *Collinsella*, *Roseburia* and Pentraxin 3 compared to the normal BMI group (see online Supplementary Table S9).

### 3.7. Multiomics Data Integration Identified Potential Biomarkers Distinguishing Urban vs. Rural Cohort

**Figure 3:**
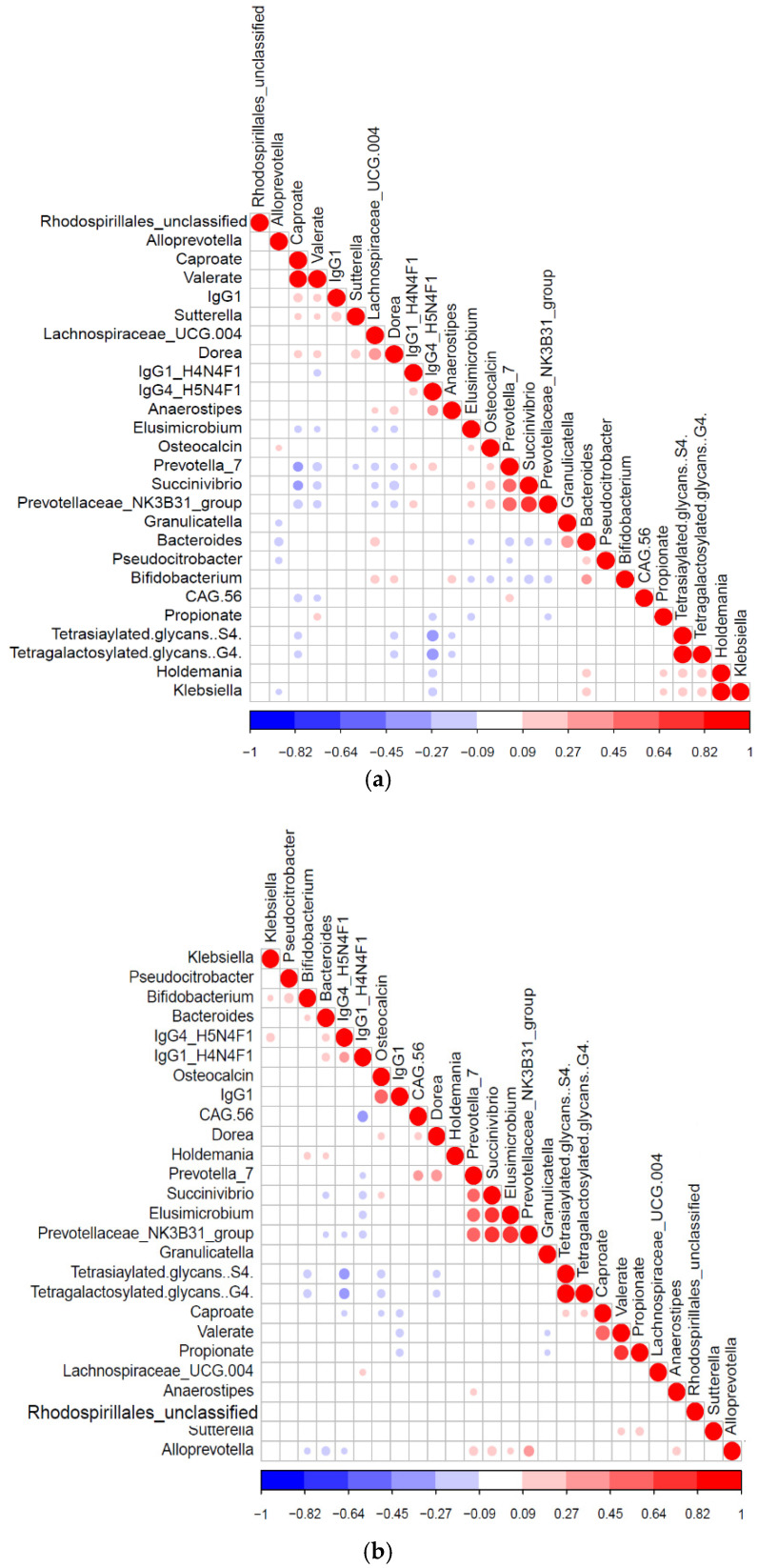
Significant Pearson correlation (*p* < 0.05) of the selected features for the (**a**) rural (*n* = 94) and (**b**) urban samples (124). Correlated variables are either highly positively correlated (in blue circles) or negatively correlated (red circles).

We constructed Pearson correlation-based heatmaps to reveal interactions between microbial taxa and immunometabolic features. These were filtered by geographic status (rural vs. urban, Figure 3). In the urban group (Figure 3B), positive associations (red circles) were seen for tetrasiaylated and tetragalactosylated serum glycans with serum caproate. Negative correlations (blue circles) were seen between these same complex glycans and *Bifidobacterium*, *Dorea*, osteocalcin and IgG4 glycopeptides with digalactosylated glycan with core fucose. Notable clusters in the rural group (Figure 3A) included positive correlations with *Holdemania* and *Klebsiella* with propionate, tetrasialylated and tetragalactosylated serum glycans.

**Figure 4:**
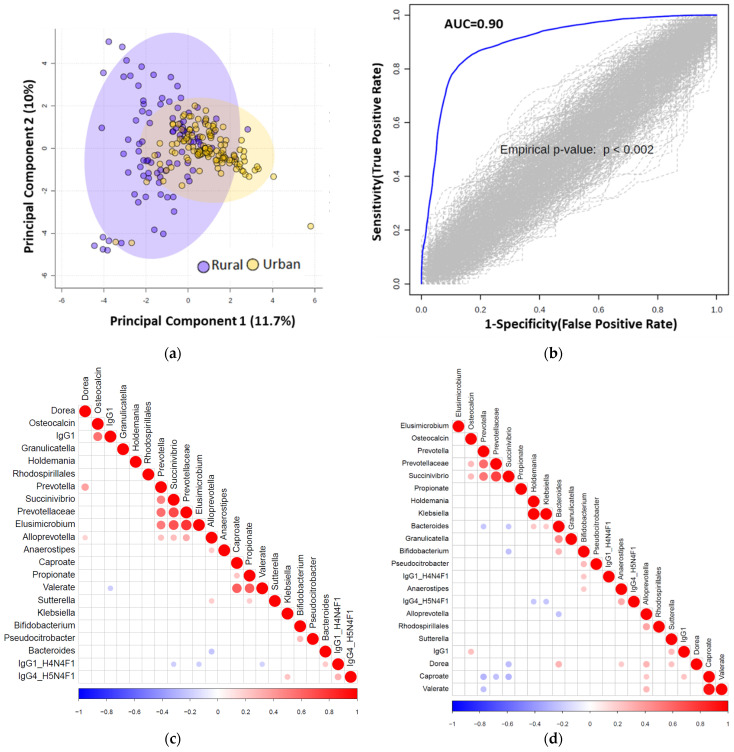
(**a**) Principal component analysis (PCA) score plot performed on the selected omics features demonstrating clustering of the rural vs. urban cohorts. Dots represent patients and are coloured according to the subject cohort. Ellipse represents 95% confidence. Results are plotted according to the Principal component-1 (PC1) and Principal component-2 (PC2) scores, with the percent variation of the cohort explained by the respective x and y axes. (**b**) Permutation test to show the stability of the AUC value after randomizing the urban and rural samples 100 times. (**c**) Significant correlation (*p* < 0.05) heatmap of the elastic net selected features is shown for urban samples. (**d**) Significant Pearson correlation (*p* < 0.05) heatmap of the elastic net selected features is shown for rural (*n* = 94) samples.

We next used elastic net (EN) algorithm to identify and select the most important features representing potential biomarker candidates distinguishing rural vs. urban (Figure 4), and normal BMI vs. overweight or pre-obese groups (see online Supplementary Figures S1 and S2). We ran the model 100 times using different training sets and ranked the selected features based on the selection frequency and chose the first quartile features. We then compared area under the curve (AUC) value with the selected features and 1000 random permuted data sets.

**Figure 5:**
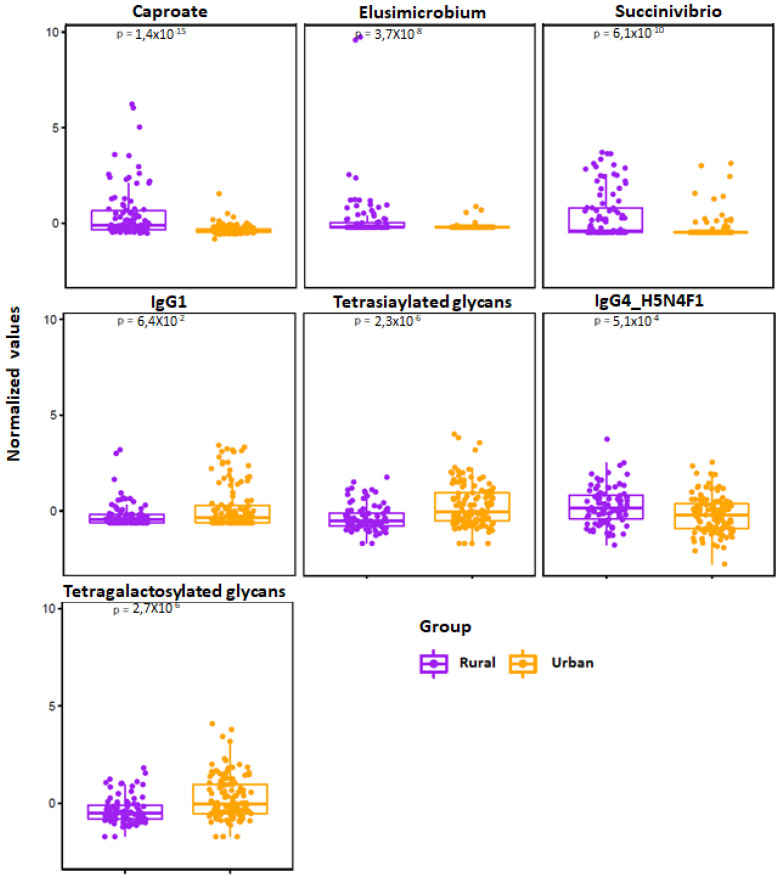
Jitter plot of the normalized selected features from elastic net analysis are shown for the rural (*n* = 94) vs. urban (*n* = 124) cohorts. Wilcoxon Rank test was performed and results were obtained, and *p*-values are shown in the respective plots.

We identified multiple distinguishing features across the rural vs. urban groups and present their frequency in online Supplementary Table S10. We show significance levels and directionality of response for identified discriminatory features in Figure 5. Using those features, we found an AUC value of 0.90 between urban vs. rural population using logistic regression (Figure 4B).

To integrate and explore associations between the selected features using the elastic net method, we generated heatmap plots (correlation method based) for urban and rural groups, as shown in Figure 4C and Figure 4D, respectively. As a representative example, the most important rural classifiers in EN were serum caproate and relative abundance of two taxa: *Elusimicrobium* and *Succinivibrio*. Caproate positively correlated with *Alloprevotella* and serum IgG1 and *Succinivibrio* with osteocalcin, *Prevotella* and *Prevotellaceae*.

For the BMI EN group comparisons, features which could distinguish between overweight and pre-obese subjects, and those with normal BMI, are displayed in online Supplementary Tables S11 and S12. PCA analysis and AUC values are displayed in Supplementary Figures S2 and S3. In particular, *Dialister* was significantly underrepresented in the pre-obese group compared to the group with normal BMI; *p* < 0.036.

## 4. Discussion

This study is the first integrative omics-based population study in which baseline gut bacterial, as well as systemic immunometabolic and glycomic traits, have been captured in geographically divergent populations in Central India. Strikingly, our findings identify a constellation of biomolecular traits that associate with metabolic dysregulation. These are principally seen in unselected urbanised populations without known diabetes. Although there is now substantial evidence connecting the gut microbiome to physiological parameters related to metabolic disorders such as diabetes [24], very few deep phenomic studies have been undertaken in Asian populations to obtain a better understanding of the biological processes associated with both healthy individuals and those at potential risk of developing diabetes. Identification of individuals at higher risk of developing diabetes is of great importance as early interventions may delay or even prevent overt diabetes. By unravelling and understanding the immunometabolic interplay between gut microbiome and the host, individualised therapeutic strategies including novel prebiotics, probiotics, synbiotics, and postbiotics could be explored to prevent or treat cardiometabolic disorders [24,43].

Several observational studies have indicated that obesity, estimated by BMI and an insulin resistance state, is a very important risk factor for T2DM [44,45,46]. Since variations in glucose metabolism are known to directly affect glycosylation, we studied serum *N*-glycan profiles and observed a more complex glycophenotype, that has previously been reported to be associated with a higher risk of developing T2DM and poorer regulation of blood sugar levels [18], in the urban population group. These pathogenic complex glycans (tetragalactosylated and tetrasialylated glycans) positively correlated with serum caproate in the urban population, and *Holdemania* and *Klebsiella* in the rural population, suggesting that these serum metabolites and genera are potentially diabetogenic. Similarly, circulating IgG *N*-glycopeptide profiles revealed a higher relative abundance of pro-inflammatory IgG glycoforms (IgG1 glycopeptides with agalactosylated glycans) in urban participants, which is consistent with that seen in other inflammatory diseases [19]. It has been suggested that agalactosylated IgG species have an enhanced capacity to activate the complement system via the lectin pathway, thereby contributing to the development of inflammation as an underlying pathological mechanism of autoimmune diseases [19]. In contrast, C -peptide, GIP, insulin and leptin correlated negatively with the anti-inflammatory gut commensal *Faecalibacterium*, a beneficial microbe which produces SCFAs [47,48,49].

Analysis of the faecal taxonomic compositional profiles revealed a dominant prevalence of *Prevotella* and *Alloprevotella* genera in rural microbiota and overrepresentation of *Bacteroides* and *Parabacteroides* in the urban microbiota, a finding substantiated by our earlier microbiome observations in Central India [50].

We observed geographic-specific variation in immunoglobulin levels, which may be due to as yet unidentified genetic and environmental factors. We surmise that frequent exposure to a wide range of infectious agents, and other environmental stressors in the rural cohort, may have skewed the humoral response towards IgM to help protect the host from invading pathogens not previously encountered. However, there is mounting evidence that natural IgM antibodies also contribute to critical innate immune functions involved in the maintenance of tissue homeostasis, including augmenting the clearance of apoptotic cells and mediating specific anti-inflammatory signaling pathways [51,52]. Higher levels of IgG in the urban cohort could reflect (meta)inflammation-associated immunosenescence, in which there is a shift towards immunoglobulins being produced by naive B cells (IgD, IgM) to immunoglobulin produced my memory B cells (IgG, IgA) [51,52]. The presence of a higher burden of diabetic-related proteins and a complex glycophenotype in the circulation of urban populations is consistent with MetS phenotype, which in younger adults may be a sign of premature ageing [53]. Thus, preventing and treating MetS and cardiovascular disease would be useful in promoting normal ageing. These findings are also in keeping with the prevalence of type 2 diabetes mellitus and metabolic syndrome which strongly associate with urban residency in Southern Asia and India, respectively [54,55]. We previously demonstrated that FMT for successful recurrent *Clostridioides difficile* associates with a reduction in the complexity of serum *N*-glycosylation profiles, which is mainly driven through a significant reduction in the relative abundance of high branching, tetragalactosylated and trisialylated glycans [56]. We therefore infer that faecal microbiota-based interventions may be useful in helping to reverse a complex glycophenotype, which may lead to improved metabolic health.

We also detected geographic-specific variation in levels of serum SCFAs which was significantly higher in the rural cohort. This particular observation has been reported in other studies where, compared to industrial human microbiomes, non-industrial gut microbiomes show greater diversity of genes involved in complex carbohydrate metabolism, and demonstrate higher amounts of SCFAs in stool [57,58]. These trends have been linked to plant-based diets rich in fibers, infrequent consumption of highly processed foods, and low exposure to pharmaceutical drugs, such as antibiotics, in non-industrialised populations [59]. Of note, we observed a positive correlation between circulating levels of IgG1 and caproate and valerate in the rural cohort, whereas a negative correlation was seen for caproate and IgG1 for the urban cohort. The former observation is supported by evidence which shows that short SCFAs function as commensal-derived stimulators of host antibody responses, by accelerating cellular metabolism and regulating gene expression to promote B cell differentiation into antibody-producing cells [60]. It remains unclear why caproate was found to negatively correlate with IgG1 in the urban population.

In terms of study limitations, not all our omics data sets were complete, a limitation which arose due to small volume of blood samples (2 mL) permitted to be collected. We were unable to acquire fasting blood samples for GSP and diabetes panel measurements and did not assess fasting blood glucose or HbA1C levels, again due to considerable practical challenges imposed by working in under-resourced and remote areas of Central India. For this same reason, we could not assess dietary or genetic effects which are likely to be important drivers of metabolic health. Moreover, we are mindful that our study findings are largely associative and not causal and, thus, will require a follow-on validation cohort study to assess their translational potential. Future work should focus on designing larger longitudinal meta-omics studies to decipher host–microbe interactions in health and disease using multi-ethnic cohorts.

## 5. Conclusions

In conclusion, we present multi-level evidence which suggests that urban living, rather than an elevated BMI, drives dysmetabolic phenotypes in young urban and rural populations in Central India. These findings start to deconvolute the complex interaction between the environment, gut microbiota, immunometabolism and dysmetabolism in a non-Western population. Our observations may serve as a launchpad for novel approaches to prediction and intervention to minimize the risk of T2DM within these vulnerable populations.

## Figures and Tables

**Table 1:** Baseline characteristics of study population. Descriptive statistics presented as the number of samples (*n*) and percentage (%) or median (interquartile range, IQR).

Characteristic	Rural, *n* = 94	Urban, *n* = 124	*p*-Value
Age, yrs (median (IQR))	39 (27, 53)	38 (30, 49)	>0.9
Gender			0.3
Female	47 (50%)	52 (42%)	
Male	47 (50%)	72 (58%)	
BMI (median (IQR))	21.0 (19.2, 22.3)	25.0 (23.5, 26.0)	<0.001
BMI Class			<0.001
Underweight	10 (11%)	0 (0%)	
Normal	68 (72%)	20 (16%)	
Overweight	12 (13%)	38 (31%)	
Pre-Obese	2 (2.1%)	62 (50%)	
Obese	2 (2.1%)	4 (3.2%)	
Smoker	23 (24%)	31 (25%)	>0.9
Hospitalized	13 (14%)	45 (36%)	
Drugs			0.017
Antacid	24 (26%)	12 (9.7%)	
PPI	1 (1.1%)	1 (0.8%)	
Co-morbidities			<0.001
Diabetes mellitus	8 (8.5%)	15 (12%)	
Epilepsy	3 (3.2%)	12 (9.7%)	
High cholesterol	0 (0%)	1 (0.8%)	
Hypertension	0 (0%)	7 (5.6%)	
Hypothyroidism	0 (0%)	1 (0.8%)	
Seizure disorder	0 (0%)	1 (0.8%)	
Tuberculosis	0 (0%)	1 (0.8%)	
Toilet facilities	80 (85%)	124 (100%)	<0.001
Hand soap	80 (85%)	124 (100%)	<0.001
Domestic animals	42 (45%)	21 (17%)	<0.001
Water supply			<0.001
Borewell	0 (0%)	18 (15%)	
Corporation water connection	6 (6.4%)	101 (81%)	
Corporation water tank	78 (83%)	3 (2.4%)	
Well water	10 (11%)	2 (1.6%)	

**Table 2:** Features which show significant differential responses between rural and urban cohorts are shown using two-tailed Student’s t-test. An FDR corrected *p*-value is shown in the last column. Arrows (↑/↓) represent features that were increased/decreased in the corresponding population.

Feature	tstat	Rural	Urban	*p*-Value (FDR Corrected)
Serum Short-chain Fatty Acids				
Caproate	6.679	↑	↓	0.000000
Valerate	5.5217	↑	↓	0.000001
Acetate	3.1602	↑	↓	0.006598
Propionate	3.0367	↑	↓	0.007375
Serum Diabetic panel				
BMI	−3.9651	↓	↑	0.003120
C-peptide	−3.4949	↓	↑	0.006466
Insulin	−3.0994	↓	↑	0.013355
Leptin	−2.9744	↓	↑	0.014119
Serum IgG Fc *N-*Glycopeptides				
IgG1 H4N4F1: IgG1 glycopeptide with monogalactosylated glycan with core fucose	−3.6748	↓	↑	0.004191
IgG4 H5N4F1: IgG4 glycopeptide with digalactosylated glycan with core fucose	3.4585	↑	↓	0.004569
IgG1 H3N4F1: IgG1 glycopeptide with agalactosylated glycan with core fucose	−2.9742	↓	↑	0.014886
IgG4 H5N4F1S1: IgG4 glycopeptide with digalactosylated and monosialylated glycan with core fucose	2.889	↑	↓	0.014886
IgG1_H5N4F1S1: IgG1 glycopeptide with digalactosylated and monosialylated glycan with core fucose.	2.5309	↑	↓	0.033823
Serum Immunoglobulin isotype				
IgG1	−3.5703	↓	↑	0.003905
IgM	2.5608	↑	↓	0.045976
Inflammation-related Protein				
IFN-γ	3.077	↑	↓	0.051323
Osteocalcin	−3.063	↓	↑	0.051323
Serum *N-*Glycans				
S4: Tetrasialylated glycans	−5.2077	↓	↑	0.000004
G4: Tetragalactosylated glycans	−5.1823	↓	↑	0.000004
AF: Antennary fucosylation	−4.7813	↓	↑	0.000019
S1: Monosialylated glycans	3.9387	↑	↓	0.000413
HB: High branching glycans	−3.9283	↓	↑	0.000413
LB: Low branching glycans	3.8475	↑	↓	0.000470
S3: Trisialylated glycans	−3.25	↓	↑	0.003435
G2: Digalactosylated glycans	2.9324	↑	↓	0.008372
G3: Trigalctosylated glycans	−2.7838	↓	↑	0.011686
B: Bisection (Glycans with bisecting GlcNAc)	2.403	↑	↓	0.030770
HM: High mannose glycans	2.2316	↑	↓	0.043612

**Table 3:** Features which demonstrate differential responses between normal and low glycated serum protein (GSP) levels (μmol/L). Low GSP = 0–199; Normal GSP = 200–285; MMP-2 = Matrix metalloproteinase-2; MMP-3 = Matrix metalloproteinase-3; sCD163 = Soluble CD163; sIL-6Rα = Soluble interleukin 6 receptor alpha; IFN-α2 = Interferon alpha-2; sCD30/TNFRSF8 = Tumour necrosis factor receptor superfamily member 8; Two-tailed Student’s t- test. An FDR corrected *p*-value is shown in the last column. Arrows (↑/↓) represent features that were increased/decreased in the corresponding population.

Feature	tstat	Normal GSP (*n* = 30)	Low GSP (*n* = 54)	*p*-Value (FDR Corrected)
MMP-2	−3.5975	↑	↓	0.000548
HM: High mannose glycans	2.8571	↓	↑	0.005416
MMP-3	2.8315	↓	↑	0.005827
sCD163	−2.7054	↑	↓	0.008297
sIL-6Rα	−2.6473	↑	↓	0.009727
IFN-α2	−2.4229	↑	↓	0.017598
IgG4 H5N4F1S1: IgG4 glycopeptide with digalactosylated and monosialylated glycan with core fucose	2.3389	↑	↓	0.021773
*Cyanobacteria*	−2.2579	↑	↓	0.026608
*Melainabacteria*	−2.2579	↑	↓	0.026608
2-methylbutyrate	−2.196	↑	↓	0.030914
AF: Antennary Fucosylation	−2.1194	↑	↓	0.03708
*Gastranaerophilales_unclassified*	−2.0844	↑	↓	0.040231
*Gastranaerophilales*	−2.0666	↑	↓	0.041926
sCD30/TNFRSF8	−2.0552	↑	↓	0.043046

**Table 4:** Features which demonstrate differential responses between normal and high GSP levels. Normal GSP = 200–285; High GSP = 286–400; APRIL/TNFSF13 = A proliferation-inducing ligand/Tumor necrosis factor ligand superfamily member, 13; Two-tailed Student’s t- test. Arrows (↑/↓) represent features that were increased/decreased in the corresponding population. An FDR corrected *p*-value is shown in the last column.

Feature	tstat	Normal GSP (*n* = 30)	High GSP (*n* = 33)	*p*-Value (FDR Corrected)
IgG2	−2.7269	↑	↓	0.008335
Caproate	−2.6832	↑	↓	0.009373
Roseburia	−2.4077	↑	↓	0.019095
Valerate	−2.2378	↑	↓	0.028897
Dorea	−2.2193	↑	↓	0.030193
IgM	−2.1594	↑	↓	0.034761
APRIL/TNFSF13	2.141	↓	↑	0.036276

**Table 5:** Features which demonstrate differential responses between normal and very high GSP levels; Normal GSP = 200–285; Very high GSP = >400. Two-tailed student’s t- test. Arrows (↑/↓) represent features that were increased/decreased in the corresponding population. An FDR corrected *p*-value is shown in the last column.

Feature	tstat	Normal GSP (*n* = 30)	Very High GSP (*n* = 18)	*p*-Value (FDR Corrected)
Caproate	2.4758	↑	↓	0.017035
Blautia	−2.0712	↓	↑	0.04398
Osteopontin	2.0162	↑	↓	0.049643

## Data Availability

Sequencing data from this study (in fastq-format) are publicly available for download at the European Nucleotide Archive (ENA) database using study accession number PRJEB42528 (http://www.ebi.ac.uk/ena/data/view/PRJEB42528 (accessed on 15 February 2021)).

## Supplementary Materials

The following are available online at https://www.mdpi.com/article/10.3390/microorganisms9071485/s1, Table S1: Sampling sites from towns and villages in rural Melghat, Amravati district, sub-divisional hospitals (SDH) and public health centres (PHC), and urban Nagpur district, Maharashtra state, India. Table S2: Study metrics (microbial and immunometabolic) Table S3: Pearson correlation coefficients correlating diabetic proteins with microbiota and molecular features in urban cohort. A corrected *p*-value was obtained from the correlation test. Table S4: Pearson correlation coefficients correlating diabetic proteins with microbiota and molecular features in rural cohort. A corrected *p*-value was obtained from the test. Table S5: Glycated serum protein levels (GSP; μmol/L). GSP levels were categorized into low, normal, high or very high categories and assessed by BMI, gender and geography. Low GSP = 0–199; Normal GSP = 200–285; High GSP = 286–400; Very high GSP = >400. BMI ranges were pre-defined using WHO Asian BMI classifications: underweight <18.5, normal (18.5–22.9), overweight (23–24.9), pre-obese (25–29.9) and obese (≥30) categories. Number of samples and percentage is represented. A chi-square test of independence was performed and a corrected *p*-value was obtained from the test. Table S6: Urban BMI group comparisons showing differential features in normal BMI vs. overweight groups. BMI ranges were pre-defined using WHO Asian BMI classifications: underweight <18.5, normal (18.5–22.9), overweight (23–24.9), pre-obese (25–29.9) and obese (≥30) categories. Median and interquartile range (IQR). Kruskal–Wallis test (for continuous data) or chi-square test of independence (for ordinal data) and corrected *p*-values shown. Table S7: Urban BMI group comparisons showing differential features in normal BMI vs. pre-obese groups. BMI ranges were pre-defined using WHO Asian BMI classifications: normal (18.5–22.9); pre-obese (25–29.9) categories. Median and interquartile range (IQR). Kruskal–Wallis test (for continuous data) or chi-square test of independence (for ordinal data) and corrected *p*-values shown. Table S8: Rural-BMI group comparisons showing differential features in normal BMI vs. pre-obese groups. BMI ranges were pre-defined using WHO Asian BMI classifications: normal (18.5–22.9); pre-obese (25–29.9) categories. Median and interquartile range (IQR). Kruskal–Wallis test (for continuous data) or chi-square test of independence (for ordinal data) and corrected *p*-values shown. Table S9: Rural-BMI group comparisons showing differential features in normal BMI vs. underweight groups. BMI ranges were pre-defined using WHO Asian BMI classifications: underweight <18.5; normal (18.5–22.9) categories. Median and interquartile range (IQR). Kruskal–Wallis test (for continuous data) or chi-square test of independence (for ordinal data) and corrected *p*-values shown. Table S10: Elastic net selected frequency of all the features for urban vs. rural group with rankings in decreasing order over 100 iterations. Table S11: Elastic net selected frequency of all the features for normal vs. overweight groups with rankings in decreasing order over 100 iterations. Table S12: Elastic net selected frequency of all the features for normal vs. pre-obese groups with rankings in decreasing order over 100 iterations. Figure S1: Log-transformed relative abundance of significantly differential genera between participants with high (≥23) or low/normal (<23) BMI score in rural and/or urban groups, as determined by Linear discriminant analysis effect size (LEfSe). *Ruminoccaceae* (group UCG-014) was significantly different in comparisons using both all samples and within urban samples alone. Figure S2: A) Principal component analysis (PCA) score plot performed on the overweight vs. normal population demonstrating clustering of subjects within samples. BMI ranges were pre-defined using WHO Asian BMI classifications: underweight normal (18.5–22.9); overweight (23–24.9) categories. PCA analysis used five selected discriminatory features (IFN-gamma, C-peptide, *Lachnospira*, *Bifidobacterium*, *Lachnoclostridium*) between normal vs. overweight using elastic net analysis. Overweight samples (in green) are more dispersed compared to the normal samples (in red). B) Area under the curve (AUC) is shown using the logistic regression method and five discriminatory features from the elastic net method. A corresponding confidence interval is also calculated as a shaded area. Figure S3: A) Principal component analysis (PCA) score plot performed on the pre-obese vs. normal population demonstrating clustering of subjects within samples. BMI ranges were pre-defined using WHO Asian BMI classifications: normal (18.5–22.9); pre-obese (25–29.9) categories. PCA analysis used twelve selected discriminatory features (*Prevotellaceae*_NK3B31_group, *Dialister*, Glucagon, C-peptide, *Prevotella*_2, Antennary fucosylation (AF), CAG-56, *Muribaculaceae*_unclassified, *Haemophilus*, *Sutterella*, *Treponema*_2, *Gastranaerophilales*_unclassified) between Normal vs. Pre-Obese using elastic net analysis. Pre-Obese (in green) and normal BMI samples (in red) seem to be separating from each other. B) Area under the curve (AUC) is shown using the logistic regression method and twelve discriminatory features from the elastic net method. A corresponding confidence interval is also calculated as a shaded area.
