# Supplementary material for: Multiomics Profiling Reveals Signatures of Dysmetabolism in Urban Populations in Central India
Source: Microorganisms. 2021 Jul 12;9(7):1485. doi: 10.3390/microorganisms9071485 (PMC8307859; doi:10.3390/microorganisms9071485)
Supplement: Supplementary file 1 [file microorganisms-09-01485-s001.zip › Monaghan 2021 supplementary information.pdf]

SUPPLEMENTARY RESULTS

A. Rural

Site Type	Name
Town/Village	Mansudhavdi
	Kutanga
	Harisal
	Jampani
	Shirpur
	Ghota
	Pohara
	Bijudhavdi
	Churakund
	Karda
	Kakarmal
	Dabyakheda
	Dabiyakheda
	Chikhli
	Zapal
	Jambu
	Tarubanda
	Keli
	Mangiya
	Rora
	Nanduri
	Chikhali
	Kara
	Kot
	Kokmar
	Katkumbh
	Chetar
	Ambadi
	Akhi
	Kadhara
Public Health Centres/Sub-	MAHAN Trust
	SDH Dharni
	PHC Bijudhavdi
	PHC Kalamkhar
	PHC Dhulghat

B. Urban

Site Type	Name
Town/Village	Ganeshpeth
	Bajaj nagar
	Shankar Nagar
	Central Bazar Road
	Sitaburdi
	Ravi Nagar
	Ramdaspeth
	Lokmat Square
	Jaripatka
	Hingna
	Wanadongri
	JafarNagar
	Sakkardara
	Dharampeth
	Medical Square
	Chattrapati Square
	Ram Nagar
	Dhantoli
	Ajni
	Panchasheel Square
	Central Avenue Road
	Darudkar Square
	Nandanwan
	Sadar
	Gokulpeth

Table S1. Sampling sites from towns and villages in rural Melghat, Amravati district, sub-divisional hospitals (SDH) and public health centres (PHC) and urban Nagpur district, Maharashtra State, India.

Category	Measure
Fecal microbiota - Phylum (relative abundance)	*Acidobacteria*
	Actinobacteria
	Bacteroidetes
	Cyanobacteria
	Deferribacteres
	Elusimicrobia
	Epsilonbacteraeota
	Firmicutes
	Fusobacteria
	Gemmatimonadetes
	Lentisphaerae
	Nitrospirae
	Proteobacteria
	Spirochaetes
	Tenericutes
	Verrucomicrobia
Fecal microbiota - Class (relative abundance)	*Acidobacteria*
	Holophagae
	Actinobacteria
	Coriobacteriia
	Bacteroidia
	Melainabacteria
	Deferribacteres
	Elusimicrobia
	Campylobacteria
	Bacilli
	Clostridia
	Erysipelotrichia
	Firmicutes_unclassified
	Negativicutes
	Fusobacteriia
	Gemmatimonadetes
	Lentisphaeria
	Nitrospira
	Alphaproteobacteria
	Deltaproteobacteria
	Gammaproteobacteria
	Proteobacteria_unclassified
	Brachyspirae
	Spirochaetia
	Mollicutes

	Verrucomicrobiae
Fecal microbiota - Order (relative abundance)	*Solibacterales*
	Holophagales
	Bifidobacteriales
	Micrococcales
	Propionibacteriales
	Coriobacteriales
	Bacteroidales
	Chitinophagales
	Sphingobacteriales
	Gastranaerophilales
	Obscuribacterales
	Deferribacterales
	Elusimicrobiales
	Campylobacterales
	Bacillales
	Lactobacillales
	Clostridiales
	Erysipelotrichales
	Firmicutes_unclassified_unclassified
	Selenomonadales
	Fusobacteriales
	Gemmatimonadales
	Victivallales
	Nitrospirales
	Alphaproteobacteria_unclassified
	Rhodospirillales
	Desulfovibrionales
	Oligoflexales
	Aeromonadales
	Betaproteobacteriales
	CHAB-XI-27
	Enterobacteriales
	Gammaproteobacteria_Incertae_Sedis
	Legionellales
	Gammaproteobacteria_unclassified
	Oceanospirillales
	Pasteurellales
	Pseudomonadales
	Salinisphaerales
	Steroidobacterales
	Proteobacteria_unclassified_unclassified

	Brachyspirales
	Spirochaetales
	Anaeroplasmatales
	Izimaplasmatales
	Mollicutes_RF39
	Verrucomicrobiales
Fecal microbiota - Family (relative abundance)	*Solibacteraceae_(Subgroup_3)*
	Holophagaceae
	Bifidobacteriaceae
	Microbacteriaceae
	Micrococcaceae
	Nocardioidaceae
	Propionibacteriaceae
	Atopobiaceae
	Coriobacteriaceae
	Eggerthellaceae
	Bacteroidaceae
	Barnesiellaceae
	Marinifilaceae
	Muribaculaceae
	Bacteroidales_unclassified
	p-2534-18B5_gut_group
	Porphyromonadaceae
	Prevotellaceae
	Rikenellaceae
	Tannerellaceae
	Chitinophagaceae
	NS11-12_marine_group
	Gastranaerophilales_unclassified
	Obscuribacterales_unclassified
	Deferribacteraceae
	Elusimicrobiaceae
	Campylobacteraceae
	Helicobacteraceae
	Family_XI
	Planococcaceae
	Staphylococcaceae
	Carnobacteriaceae
	Enterococcaceae
	Lactobacillaceae
	Leuconostocaceae
	Streptococcaceae

	Christensenellaceae
	Clostridiaceae_1
	Clostridiales_vadinBB60_group
	Family_XIII
	Lachnospiraceae
	Clostridiales_unclassified
	Peptostreptococcaceae
	Ruminococcaceae
	Erysipelotrichaceae
	Firmicutes_unclassified_unclassified_unclassified
	Acidaminococcaceae
	Veillonellaceae
	Fusobacteriaceae
	Leptotrichiaceae
	Gemmatimonadaceae
	vadinBE97
	Victivallaceae
	Nitrospiraceae
	Alphaproteobacteria_unclassified_unclassified
	Rhodospirillales_unclassified
	Desulfovibrionaceae
	0319-6G20
	Aeromonadaceae
	Succinivibrionaceae
	Burkholderiaceae
	Betaproteobacteriales_unclassified
	Neisseriaceae
	Nitrosomonadaceae
	CHAB-XI-27_unclassified
	Enterobacteriaceae
	Unknown_Family
	Legionellaceae
	Gammaproteobacteria_unclassified_unclassified
	Halomonadaceae
	Pasteurellaceae
	Moraxellaceae
	Pseudomonadaceae
	Solimonadaceae
	Steroidobacteraceae
	Proteobacteria_unclassified_unclassified_unclassified
	Brachyspiraceae
	Spirochaetaceae

	Anaeroplasmataceae
	Izimaplasmatales_unclassified
	Mollicutes_RF39_unclassified
	Akkermansiaceae
Fecal microbiota - Genus (relative abundance)	*Bryobacter*
	Geothrix
	Bifidobacterium
	Lysinimonas
	Microbacterium
	Pseudoglutamicibacter
	Rothia
	Marmoricola
	Cutibacterium
	Libanicoccus
	Olsenella
	Collinsella
	Adlercreutzia
	Eggerthella
	Senegalimassilia
	Slackia
	Bacteroides
	Barnesiella
	Odoribacter
	Muribaculaceae_unclassified
	Bacteroidales_unclassified_unclassified
	p-2534-18B5_gut_group_unclassified
	Porphyromonas
	Alloprevotella
	Prevotellaceae_unclassified
	Paraprevotella
	Prevotella_1
	Prevotella_2
	Prevotella_7
	Prevotella_9
	Prevotellaceae_NK3B31_group
	Prevotellaceae_UCG-001
	Alistipes
	Rikenellaceae_RC9_gut_group
	Macellibacteroides
	Parabacteroides
	Asinibacterium
	Chitinophagaceae_unclassified

	Sediminibacterium
	NS11-12_marine_group_unclassified
	Gastranaerophilales_unclassified_unclassified
	Obscuribacterales_unclassified_unclassified
	Mucispirillum
	Elusimicrobium
	Campylobacter
	Helicobacter
	Gemella
	Psychrobacillus
	Staphylococcus
	Granulicatella
	Enterococcus
	Lactobacillus
	Weissella
	Streptococcus
	Christensenellaceae_R-7_group
	Candidatus_Arthromitus
	Clostridium_sensu_stricto_1
	Clostridiales_vadinBB60_group_unclassified
	Family_XIII_AD3011_group
	Family_XIII_UCG-001
	Agathobacter
	Anaerostipes
	Blautia
	Butyrivibrio
	CAG-56
	Coprococcus_1
	Coprococcus_2
	Coprococcus_3
	Dorea
	GCA-900066755
	Hungatella
	Lachnoclostridium
	Lachnospira
	Lachnospiraceae_AC2044_group
	Lachnospiraceae_ND3007_group
	Lachnospiraceae_NK4A136_group
	Lachnospiraceae_UCG-001
	Lachnospiraceae_UCG-004
	Lachnospiraceae_unclassified
	Oribacterium

	Roseburia
	Tyzzerella
	Tyzzerella_3
	Tyzzerella_4
	Clostridiales_unclassified_unclassified
	Clostridioides
	Intestinibacter
	Romboutsia
	Anaerotruncus
	Butyricicoccus
	DTU089
	Faecalibacterium
	Flavonifractor
	Fournierella
	Ruminococcaceae_unclassified
	Oscillibacter
	Oscillospira
	Ruminiclostridium_5
	Ruminiclostridium_6
	Ruminiclostridium_9
	Ruminococcaceae_NK4A214_group
	Ruminococcaceae_UCG-002
	Ruminococcaceae_UCG-003
	Ruminococcaceae_UCG-005
	Ruminococcaceae_UCG-009
	Ruminococcaceae_UCG-010
	Ruminococcaceae_UCG-013
	Ruminococcaceae_UCG-014
	Ruminococcus_1
	Ruminococcus_2
	Subdoligranulum
	UBA1819
	Asteroleplasma
	Catenibacterium
	Dielma
	Erysipelatoclostridium
	Erysipelotrichaceae_UCG-003
	Erysipelotrichaceae_UCG-004
	Holdemanella
	Holdemania
	Erysipelotrichaceae_unclassified
	Solobacterium

	FIrmicutes_unclassified_unclassified_unclassified
	Acidaminococcus
	Phascolarctobacterium
	Allisonella
	Anaerovibrio
	Dialister
	Megamonas
	Megasphaera
	Mitsuokella
	Veillonellaceae_unclassified
	Selenomonas_1
	Veillonella
	Fusobacterium
	Leptotrichia
	Gemmatimonas
	vadinBE97_unclassified
	Victivallis
	Nitrospira
	Alphaproteobacteria_unclassified_unclassified_unclassified
	Rhodospirillales_unclassified_unclassified
	Bilophila
	Desulfovibrionaceae_unclassified
	0319-6G20_unclassified
	Aeromonas
	Anaerobiospirillum
	Succinivibrio
	Achromobacter
	Aquabacterium
	Comamonas
	Curvibacter
	Delftia
	Oxalobacter
	Parasutterella
	Pelomonas
	Ralstonia
	Rhizobacter
	Sutterella
	Variovorax
	Betaproteobacteriales_unclassified_unclassified
	Neisseria
	IS-44
	MND1

	CHAB-XI-27_unclassified_unclassified
	Citrobacter
	Escherichia/Shigella
	Hafnia-Obesumbacterium
	Klebsiella
	Enterobacteriaceae_unclassified
	Proteus
	Pseudocitrobacter
	Raoultella
	Salmonella
	Yokenella
	Acidibacter
	Legionella
	Gammaproteobacteria_unclassified_unclassified_unclassified
	Halomonas
	Haemophilus
	Acinetobacter
	Pseudomonas
	Alkanibacter
	Steroidobacteraceae_unclassified
	Proteobacteria_unclassified_unclassified_unclassified_unclassified
	Brachyspira
	Treponema_2
	Anaeroplasma
	Izimaplasmatales_unclassified_unclassified
	Mollicutes_RF39_unclassified_unclassified
	Akkermansia
Short chain fatty acids (relative abundance)	2-hydroxybutyrate
	2-methylbutyrate
	Acetate
	Butyrate
	Caproate
	Isobutyrate
	Isovalerate
	Propionate
	Valerate
Cytokine/chemokine panel (pg/mL)	APRIL/TNFSF13
	BAFF/TNFSF13B
	sCD30/TNFRSF8
	sCD163
	Chitinase 3-like 1
	gp130/sIL-6Rb
	IFN-a2

	IFN-b
	IFN-gamma
	IL-2
	sIL-6Ra
	IL-8
	IL-10
	IL-11
	IL-12 (p40)
	IL-12 (p70)
	IL-19
	IL-20
	IL-22
	IL-26
	IL-27 (p28)
	IL-28A/IFN-lamda2
	IL-29/IFN-lamda1
	IL-32
	IL-34
	IL-35
	LIGHT/TNFSF14
	MMP-1
	MMP-2
	MMP-3
	Osteocalcin
	Osteopontin (OPN)
	Pentraxin-3
	sTNF-R1
	sTNF-R2
	TSLP
	TWEAK/TNFSF12
Multi-isotype panel (ng/mL)	IgG2
	IgG3
	IgM
	IgG1
	IgG4
	IgA
Diabetes panel (pg/mL)	C-peptide
	Ghrelin
	GIP
	GLP-1
	Glucagon
	Insulin
	Leptin
	PAI-1
	Resistin

	Visfatin
Glycated Serum Protein (micromol/L)	GSP
Serum N-glycan traits (relative abundance)	Low-branching glycans (LB)
	High-branching glycans (HB)
	Neutral (not siaylated) glycans (S0)
	Monosiaylated glycans (S1)
	Disiaylated glycans (S2)
	Trisiaylated glycans (S3)
	Tetrasiaylated glycans (S4)
	Agalactosylated glycans (G0)
	Monogalactosylated glycans (G1)
	Digalactosylated glycans (G2)
	Trigalactosylated glycans (G3)
	Tetragalactosylated glycans (G4)
	Highmannose glycans (HM)
	Bisection (Glycans with bisecting GlcNAc) (B)
	Core fucosylation (CF)
	Antennary fucosylation (AF)
IgG Fc N-glycan traits (relative abundance)	IgG1 glycopeptide with agalactosylated glycan
	IgG1 glycopeptide with monogalactosylated glycan
	IgG1 glycopeptide with digalactosylated glycan
	IgG1 glycopeptide with digalactosylated and monosialylated glycan
	IgG2&3 glycopeptides with agalactosylated glycan
	IgG2&3 glycopeptide with monogalactosylated glycan
	IgG2&3 glycopeptides with digalactosylated glycan
	IgG2&3 glycopeptides with digalactosylated and monosialylated glycan
	IgG4 glycopeptide with agalactosylated glycan
	IgG4 glycopeptides with monogalactosylated glycan
	IgG4 glycopeptide with digalactosylated glycan
	IgG4 glycopeptide with digalactosylated and monosialylated glycan

Table S2. Study metrics (microbial and immunometabolic).

Feature 1	Feature 2	Pearson Correlation coefficient	p-value
Marinifilaceae	Visfatin	0.96577706	0.0000000
Odoribacter	Visfatin	0.96577706	0.0000000
Erysipelatoclostridium	Visfatin	0.96577706	0.0000000
Bacillales	Visfatin	0.96577706	0.0000000
Staphylococcaceae	Visfatin	0.96577706	0.0000000
Macellibacteroides	Visfatin	0.96577706	0.0000000
Staphylococcus	Visfatin	0.96577706	0.0000000
Hungatella	Visfatin	0.96577706	0.0000000
Ruminiclostridium_6	Visfatin	0.96577706	0.0000000
Acidaminococcus	Visfatin	0.96577706	0.0000000
Lactobacillaceae	Visfatin	0.9651265	0.0000000
Erysipelotrichia	Visfatin	0.95713275	0.0000000
Erysipelotrichales	Visfatin	0.95713275	0.0000000
Erysipelotrichaceae	Visfatin	0.95713275	0.0000000
Lactobacillus	Visfatin	0.95592119	0.0000000
Erysipelotrichaceae_unclassified	Visfatin	0.95428688	0.0000000
Lachnoclostridium	Ghrelin	0.9206094	0.0000011
Actinobacteria.1	Leptin	0.90835518	0.0000028
Actinobacteria	Leptin	0.90622177	0.0000033
Klebsiella	Ghrelin	0.90568506	0.0000034
Bifidobacteriales	Leptin	0.90468769	0.0000036
Bifidobacteriaceae	Leptin	0.90468769	0.0000036
Bifidobacterium	Leptin	0.90468769	0.0000036
Escherichia/Shigella	GLP-1	0.81351334	0.0002249
Enterobacteriales	GIP	0.80631514	0.0002824
Enterobacteriaceae	GIP	0.80631514	0.0002824
Enterobacteriales	GLP-1	0.80573778	0.0002875
Enterobacteriaceae	GLP-1	0.80573778	0.0002875
Gammaproteobacteria	GIP	0.79948827	0.0003475
Proteobacteria	GIP	0.7967379	0.0003770
Proteobacteria	GLP-1	0.78411827	0.0005397
Gammaproteobacteria	GLP-1	0.78345866	0.0005496
Osteocalcin	Insulin	0.76074577	0.0009904
Escherichia/Shigella	Leptin	0.7575692	0.0010700
Escherichia/Shigella	GIP	0.75599903	0.0011113
Enterobacteriales	Leptin	0.72709012	0.0021311
Enterobacteriaceae	Leptin	0.72709012	0.0021311
Actinobacteria	GLP-1	0.7151733	0.0027253
Osteocalcin	GLP-1	0.71384355	0.0027991
Bifidobacteriales	GLP-1	0.71366136	0.0028093

Bifidobacteriaceae	GLP-1	0.71366136	0.0028093
Bifidobacterium	GLP-1	0.71366136	0.0028093
Escherichia/Shigella	Insulin	0.69419199	0.0040880
Actinobacteria	GIP	0.69336277	0.0041513
Bifidobacteriales	GIP	0.69215244	0.0042450
Bifidobacteriaceae	GIP	0.69215244	0.0042450
Bifidobacterium	GIP	0.69215244	0.0042450
Osteocalcin	GIP	0.6865928	0.0046974
Proteobacteria	Leptin	0.68007984	0.0052751
Gammaproteobacteria	Leptin	0.68006865	0.0052761
Gammaproteobacteria	C-peptide	0.67413683	0.0058499
Proteobacteria	C-peptide	0.67345576	0.0059188
Enterobacteriales	C-peptide	0.671603	0.0061096
Enterobacteriaceae	C-peptide	0.671603	0.0061096
IgG1	C-peptide	0.65323301	0.0082751
Escherichia/Shigella	C-peptide	0.64772893	0.0090289
APRIL/TNFSF13	Ghrelin	0.64186243	0.0098904
Actinobacteria	Insulin	0.63620936	0.0107800
Osteocalcin	C-peptide	0.63225941	0.0114377
Bifidobacteriales	Insulin	0.63041148	0.0117559
Bifidobacteriaceae	Insulin	0.63041148	0.0117559
Bifidobacterium	Insulin	0.63041148	0.0117559
Enterobacteriales	Insulin	0.62929453	0.0119515
Enterobacteriaceae	Insulin	0.62929453	0.0119515
Gammaproteobacteria	Insulin	0.6226059	0.0131767
Proteobacteria	Insulin	0.62034049	0.0136129
IgG1	GLP-1	0.59847744	0.0184221
Oxalobacter	C-peptide	0.59841554	0.0184373
Megasphaera	Visfatin	0.59789522	0.0185659
Tannerellaceae	PAI-1	-0.5972789	0.0187190
IgA	Glucagon	-0.6106287	0.0156114
Parabacteroides	PAI-1	-0.6207857	0.0135263
Lachnospiraceae_UCG.001	PAI-1	-0.6314116	0.0115828
Ruminococcaceae_UCG.013	PAI-1	-0.637238	0.0106136
Flavonifractor	PAI-1	-0.6413111	0.0099745
Veillonella	PAI-1	-0.6803338	0.0052515
MMP-1	Glucagon	-0.6983991	0.0037787
Bacteroidaceae	PAI-1	-0.703926	0.0034010
Bacteroides	PAI-1	-0.703926	0.0034010

Table S3. Pearson correlation coefficients correlating diabetic proteins with microbiota and molecular features in urban cohort. A corrected p-value was obtained from the correlation test.

Feature 1	Feature 2	Pearson Correlation Coefficient	p-value
Erysipelotrichaceae_unclassified	GLP-1	0.946279	0.000000
Anaeroplasmatales	GLP-1	0.945789	0.000000
Anaeroplasmataceae	GLP-1	0.945789	0.000000
Erysipelotrichaceae_UCG.004	GLP-1	0.945789	0.000000
Anaeroplasma	GLP-1	0.945789	0.000000
Asteroleplasma	GLP-1	0.921651	0.000001
Paraprevotella	C-peptide	0.904203	0.000004
Flavonifractor	C-peptide	0.902171	0.000004
UBA1819	C-peptide	0.902171	0.000004
Erysipelatoclostridium	C-peptide	0.902171	0.000004
Clostridiaceae_1	GIP	0.901197	0.000005
Clostridium_sensu_stricto_1	GIP	0.901197	0.000005
Alistipes	C-peptide	0.896953	0.000006
Bacteroidaceae	C-peptide	0.892468	0.000008
Bacteroides	C-peptide	0.892468	0.000008
Mollicutes_RF39_unclassified	GIP	0.886008	0.000011
IL-32	Visfatin	0.878882	0.000016
Tannerellaceae	C-peptide	0.878477	0.000016
Parabacteroides	C-peptide	0.878477	0.000016
Bacteroidales_unclassified	GIP	0.875453	0.000019
IL-34	Visfatin	0.865991	0.000030
Ruminococcaceae_UCG.013	GIP	0.820114	0.000181
IFN-α2	Visfatin	0.817993	0.000194
IL-19	Visfatin	0.816809	0.000202
IL-10	Visfatin	0.816567	0.000204
IL-2	Visfatin	0.811361	0.000241
Firmicutes_unclassified	Visfatin	0.810318	0.000249
Victivallaceae	Visfatin	0.810318	0.000249
Victivallis	Visfatin	0.810318	0.000249
Parasutterella	Visfatin	0.810318	0.000249
Proteobacteria_unclassified	Visfatin	0.807220	0.000275
Lentisphaerae	Visfatin	0.805650	0.000288
Lentisphaeria	Visfatin	0.805650	0.000288
Victivallales	Visfatin	0.805650	0.000288
vadinBE97_unclassified	Visfatin	0.805127	0.000293
vadinBE97	Visfatin	0.803941	0.000304
Ruminococcaceae_UCG.010	Visfatin	0.790944	0.000446
IL-26	Visfatin	0.787574	0.000490
sCD163	Visfatin	0.785398	0.000521

IL-8	Visfatin	0.781605	0.000578
Cyanobacteria	Visfatin	0.781594	0.000578
Melainabacteria	Visfatin	0.781594	0.000578
Gastranaerophilales	Visfatin	0.781594	0.000578
Gastranaerophilales_unclassified	Visfatin	0.781594	0.000578
Tenericutes	GIP	0.781507	0.000580
Mollicutes	GIP	0.781507	0.000580
Mollicutes_RF39	GIP	0.779689	0.000609
sTNF-R1	Visfatin	0.777808	0.000640
sCD30/TNFRSF8	Visfatin	0.768987	0.000806
IL-35	Visfatin	0.767972	0.000827
Family_XIII_AD3011_group	Visfatin	0.761122	0.000981
Ruminococcaceae_UCG.005	Visfatin	0.760266	0.001002
Elusimicrobia	Visfatin	0.758333	0.001050
Elusimicrobiales	Visfatin	0.758333	0.001050
Elusimicrobiaceae	Visfatin	0.758333	0.001050
Elusimicrobium	Visfatin	0.758333	0.001050
IL-12 (p70)	Visfatin	0.748910	0.001314
Prevotellaceae_NK3B31_group	Visfatin	0.746798	0.001380
BAFF/TNFSF13B	Visfatin	0.742469	0.001523
Family_XIII	Visfatin	0.742398	0.001525
Aeromonadales	Visfatin	0.741031	0.001573
IL-12 (p40)	Visfatin	0.726045	0.002179
Christensenellaceae	GIP	0.689723	0.004438
Christensenellaceae_R.7_group	GIP	0.689723	0.004438
sTNF-R2	Visfatin	0.688313	0.004554
Mitsuokella	GLP-1	0.685639	0.004779
Ruminococcaceae_UCG.014	GIP	0.677069	0.005560
Lactobacillus	GIP	0.672482	0.006019
Acidaminococcaceae	GIP	0.669790	0.006301
Izimaplasmatales	Visfatin	0.668802	0.006407
Izimaplasmatales_unclassified	Visfatin	0.668802	0.006407
LIGHT/TNFSF14	Visfatin	0.665504	0.006772
Megasphaera	Leptin	0.661988	0.007179
Ruminococcaceae_UCG.003	Visfatin	0.661770	0.007204
Rikenellaceae_RC9_gut_group	Visfatin	0.655954	0.007921
Acidaminococcus	GIP	0.654547	0.008103
Lactobacillaceae	GIP	0.639729	0.010219
Roseburia	Insulin	0.639181	0.010305
Faecalibacterium	C-peptide	0.637319	0.010601
MMP-2	Visfatin	0.636929	0.010663
Clostridiales_vadinBB60_group	Visfatin	0.635279	0.010932

Clostridiales_vadinBB60_group_unclassified	Visfatin	0.635279	0.010932
Ruminococcaceae_NK4A214_group	GIP	0.630584	0.011726
Prevotella_7	GLP-1	0.627370	0.012295
Pentraxin 3	Visfatin	0.622840	0.013132
Rikenellaceae	Visfatin	0.622493	0.013198
Lachnospiraceae_UCG.001	Leptin	0.618143	0.014047
IL-22	Visfatin	0.615007	0.014684
gp130/sIL-6Rβ	Visfatin	0.611390	0.015447
Succinivibrionaceae	Visfatin	0.610606	0.015616
Succinivibrio	Visfatin	0.610606	0.015616
Dorea	C.peptide	0.610370	0.015667
Proteobacteria	Visfatin	0.603265	0.017272
Coprococcus_2	Leptin	0.601479	0.017694
Roseburia	Leptin	0.598913	0.018315
Firmicutes	Insulin	0.596158	0.019000
Flavonifractor	Ghrelin	-0.593822	0.019596
UBA1819	Ghrelin	-0.593822	0.019596
Erysipelatoclostridium	Ghrelin	-0.593822	0.019596
Bacteroidaceae	Glucagon	-0.597932	0.018557
Bacteroides	Glucagon	-0.597932	0.018557
Bacteroidaceae	Resistin	-0.602860	0.017367
Bacteroides	Resistin	-0.602860	0.017367
Bacteroidaceae	Ghrelin	-0.605275	0.016806
Bacteroides	Ghrelin	-0.605275	0.016806
Paraprevotella	Resistin	-0.607853	0.016222
2-hydroxybutyrate	PAI-1	-0.608135	0.016159
Flavonifractor	Resistin	-0.608425	0.016095
UBA1819	Resistin	-0.608425	0.016095
Erysipelatoclostridium	Resistin	-0.608425	0.016095
Isobutyrate	C.peptide	-0.613163	0.015069
Dorea	Resistin	-0.614460	0.014798
Dorea	Ghrelin	-0.616486	0.014381
Burkholderiaceae	Glucagon	-0.619764	0.013726
Tannerellaceae	Resistin	-0.629082	0.011989
Parabacteroides	Resistin	-0.629082	0.011989
Tannerellaceae	Ghrelin	-0.662779	0.007085
Parabacteroides	Ghrelin	-0.662779	0.007085
Betaproteobacteriales	Glucagon	-0.662965	0.007064
Tannerellaceae	Glucagon	-0.673043	0.005961
Parabacteroides	Glucagon	-0.673043	0.005961
Faecalibacterium	Resistin	-0.679080	0.005369
Deltaproteobacteria	Glucagon	-0.710918	0.002967

Desulfovibrionales	Glucagon	-0.710918	0.002967
Desulfovibrionaceae	Glucagon	-0.710918	0.002967
Bilophila	Glucagon	-0.710918	0.002967

Table S4. Pearson correlation coefficients correlating diabetic proteins with microbiota and molecular features in rural cohort. A corrected p-value was obtained from the test.

Characteristic	GSP				p-value
	**Low**, n = 54	**Normal**, n = 30	**High**, n = 33	**Very High**, n = 18	
**BMI**					<0.001
Underweight	6 (11%)	0 (0%)	1 (3.1%)	0 (0%)	
Normal	31 (58%)	18 (60%)	4 (12%)	2 (11%)	
Overweight	10 (19%)	3 (10%)	11 (34%)	2 (11%)	
Pre-obese	5 (9.4%)	9 (30%)	15 (47%)	13 (72%)	
Obese	1 (1.9%)	0 (0%)	1 (3.1%)	1 (5.6%)	
**Gender**					0.8
Female	25 (47%)	16 (53%)	14 (44%)	7 (39%)	
Male	28 (53%)	14 (47%)	18 (56%)	11 (61%)	
**Geography**					<0.001
Rural	36 (68%)	16 (53%)	5 (16%)	1 (5.6%)	
Urban	17 (32%)	14 (47%)	27 (84%)	17 (94%)	

Table S5. Glycated serum protein levels (GSP; μmol/L). GSP levels were categorised into low, normal, high or very high categories and assessed by BMI, gender and geography. Low GSP = 0-199; Normal GSP = 200-285; High GSP = 286-400; Very high GSP = >400. BMI ranges were pre-defined using WHO Asian BMI classifications: underweight <18.5, normal (18.5-22.9), overweight (23-24.9), pre-obese (25-29.9) and obese (≥30) categories. Number of samples and percentage is represented. A chi-square test of independence was performed and a corrected p-value was obtained from the test.

**Characteristic**	**Normal**, N = 31	**Overweight**, N = 26	**P value**
2-hydroxybutyrate	356,778 (206,099, 497,945)	511,760 (293,382, 1,173,990)	0.014
Isobutyrate	193,094 (177,967, 211,729)	229,356 (189,306, 341,504)	0.045
Propionate	901,293 (842,112, 1,015,544)	990,890 (915,415, 1,111,326)	0.012
Valerate	17,263 (16,150, 19,361)	20,628 (17,792, 22,707)	0.008
IgM	1.99 (1.33, 2.73)	0.94 (0.67, 1.94)	0.049

Table S6. Urban-BMI group comparisons showing differential features in normal BMI vs overweight groups. BMI ranges were pre-defined using WHO Asian BMI classifications: underweight <18.5, normal (18.5-22.9), overweight (23-24.9), pre-obese (25-29.9) and obese (≥30) categories. Median and interquartile range (IQR). Kruskal-Wallis test (for continuous data) or chi-square test of independence (for ordinal data) and corrected p values shown.

**Characteristic**	**Normal**, N = 31	**Pre-obese**, N = 37	**P value**
Acetate	3,340,416 (2,949,719, 3,973,637)	4,005,463 (3,196,825, 5,703,065)	0.037
Isobutyrate	193,094 (177,967, 211,729)	228,114 (199,638, 279,733)	0.006
Propionate	901,293 (842,112, 1,015,544)	987,172 (914,587, 1,120,897)	0.026
Valerate	17,263 (16,150, 19,361)	20,063 (17,595, 22,071)	0.042
IgM	1.99 (1.33, 2.73)	1.21 (0.70, 1.74)	0.007

Table S7. Urban-BMI group comparisons showing differential features in normal BMI vs pre-obese groups. BMI ranges were pre-defined using WHO Asian BMI classifications: normal (18.5-22.9); pre-obese (25-29.9) categories. Median and interquartile range (IQR). Kruskal-Wallis test (for continuous data) or chi-square test of independence (for ordinal data) and corrected p values shown.

**Characteristic**	**Normal**, N = 37	**Pre-obese**, N = 19	**P value**
Collinsella	0.0 (0.0, 5.1)	3.6 (0.0, 8.9)	0.048
Bacteroides	4 (1, 35)	0 (0, 3)	0.006
Prevotella_9	235 (118, 335)	319 (269, 349)	0.041
Agathobacter	13 (0, 34)	81 (28, 125)	0.002
Roseburia	9 (0, 33)	39 (24, 46)	0.004
Faecalibacterium	46 (20, 73)	82 (50, 119)	0.032
Ruminococcaceae_unclassified	1 (0, 3)	5 (1, 19)	0.016
Ruminococcaceae_UCG_014	0.0 (0.0, 3.7)	3.1 (1.2, 5.0)	0.019
Catenibacterium	1 (0, 9)	7 (3, 16)	0.035
Megasphaera	0.0 (0.0, 0.0)	2.2 (0.0, 7.6)	0.003
Mitsuokella	0 (0, 3)	9 (0, 15)	0.003
2-methylbutyrate	103,443 (35,990, 186,737)	23,504 (19,941, 39,587)	0.003
Acetate	5,580,045 (4,430,044, 9,151,783)	4,019,140 (3,089,731, 5,176,548)	0.008
Caproate	108,095 (75,414, 166,538)	70,149 (55,557, 118,449)	0.014
Isobutyrate	339,622 (253,411, 439,625)	222,050 (196,492, 258,262)	0.005
Isovalerate	261,035 (74,897, 444,494)	53,548 (26,097, 75,369)	0.004

Table S8. Rural-BMI group comparisons showing differential features in normal BMI vs pre-obese groups. BMI ranges were pre-defined using WHO Asian BMI classifications: normal (18.5-22.9); pre-obese (25-29.9) categories. Median and interquartile range (IQR). Kruskal-Wallis test (for continuous data) or chi-square test of independence (for ordinal data) and corrected p values shown.

Characteristic	**Normal**, N = 37	**Underweight, N = 8**	**P value**
Collinsella	0.0 (0.0, 5.1)	3.5 (1.7, 13.5)	0.019
Prevotella_7	3 (0, 42)	0 (0, 0)	0.04
Roseburia	9 (0, 33)	35 (21, 60)	0.037
Pentraxin 3	8,584 (4,851, 17,464)	26,696 (21,793, 40,852)	0.036

Table S9. Rural-BMI group comparisons showing differential features in normal BMI vs underweight groups. BMI ranges were pre-defined using WHO Asian BMI classifications: underweight <18.5; normal (18.5-22.9) categories. Median and interquartile range (IQR). Kruskal-Wallis test (for continuous data) or chi-square test of independence (for ordinal data) and corrected p values shown.

Selected features	Frequency
Caproate	100
Elusimicrobium	100
Succinivibrio	100
G4: Tetragalactosylated.glycans	100
S4: Tetrasialylated.glycans	100
IgG1	99
Osteocalcin	98
Prevotella_7	94
Lachnospiraceae_UCG.004	92
IgG4 H5N4F1: IgG4 glycopeptide with digalactosylated glycan with core fucose	91
Pseudocitrobacter	88
IgG1 H4N4F1: IgG1 glycopeptide with monogalactosylated glycan with core fucose	85
Klebsiella	79
Prevotellaceae_NK3B31_group	77
CAG-56	76
Valerate	76
Granulicatella	71
Bacteroides	70
Propionate	70
Sutterella	69
Bifidobacterium	68
Alloprevotella	67
Anaerostipes	67
Dorea	67
Holdemania	66
Ruminococcaceae_UCG.014	63
Holdemanella	62
IFN-γ	61
IgG1 H5N4F1S1	61
S1: Monosialylated glycans	61
Escherichia/Shigella	59
Solobacterium	59
Rhodospirillales_unclassified	56
Paraprevotella	55
Streptococcus	55
Roseburia	54
Weissella	53
Christensenellaceae_R.7_group	44
AF: Antennary fucosylation	43
Mitsuokella	42
Rikenellaceae_RC9_gut_group	42

IgG4 H4N4F1: IgG4 glycopeptide with monogalactosylated glycan with core fucose	40
Clostridiales_vadinBB60_group_unclassified	39
Alistipes	38
Bacteroidales_unclassified	38
IgA	38
Megamonas	37
Ruminococcaceae_UCG.009	36
IL-28A/IFN-λ2	34
Acinetobacter	33
G2: Digalactosylated glycans	33
Enterobacteriaceae_unclassified	33
IgM	33
Selenomonas_1	32
Ralstonia	31
IL-29/IFN-λ1	30
Olsenella	30
Ruminococcus_1	30
Barnesiella	29
Oribacterium	29
Fournierella	28
IgG4 H5N4F1S1: IgG4 glycopeptide with digalactosylated and monosialylated glycan with core fucose	28
Allisonella	26
S2: Disialylated glycans	26
Coprococcus_3	25
Fusobacterium	25
2-hydroxybutyrate	25
Lactobacillus	22
MMP-3	22
DTU089	21
Tyzzerella_4	21
Intestinibacter	20
Ruminococcaceae_UCG.010	20
APRIL/TNFSF13	19
Romboutsia	19
Curvibacter	18
Dialister	18
MMP-2	18
Lysinimonas	17
Subdoligranulum	17
IgG2 H3N4F1: IgG2&3 glycopeptides with agalactosylated glycan with core fucose	16
IgG2 H5N4F1S1: IgG2&3 glycopeptides with digalactosylated and monosialylated glycan with core fucose	16

Proteobacteria_unclassified	16
Aeromonas	15
Anaerovibrio	15
Brachyspira	15
Clostridioides	15
Veillonellaceae_unclassified	15
Haemophilus	14
Odoribacter	14
Ruminococcaceae_UCG.003	14
Agathobacter	13
IL-12 (p70)	13
vadinBE97_unclassified	13
Erysipelotrichaceae_UCG.004	12
Gastranaerophilales_unclassified	12
IgG2_H4N4F1: IgG2&3 glycopeptides with monogalactosylated glycan with core fucose	12
Megasphaera	12
Prevotella_2	12
Bilophila	11
B: Bisection (Glycans with bisecting GlcNAc)	11
IL-8	11
Oscillospira	11
Treponema_2	11
Veillonella	11
Erysipelatoclostridium	10
Obscuribacterales_unclassified	10
Proteus	10
sTNF-R1	10
UBA1819	10
Enterococcus	9
Gammaproteobacteria_unclassified	9
Isobutyrate	9
Parabacteroides	9
G3: Trigalactosylated glycans	9
Gemmatimonas	8
IL-27 (p28)	8
MND1	8
G1: Monogalactosylated glycans	8
Victivallis	8
Catenibacterium	7
Clostridium_sensu_stricto_1	7
IFN-α2	7
Aquabacterium	6
Blautia	6
Bryobacter	6

Collinsella	6
Lachnoclostridium	6
Lachnospiraceae_AC2044_group	6
Achromobacter	5
Erysipelotrichaceae_UCG.003	5
Flavonifractor	5
IgG4	5
IL-32	5
Lachnospiraceae_ND3007_group	5
LB: Low branching glycans	5
Mollicutes_RF39_unclassified	5
Pentraxin 3	5
Prevotellaceae_unclassified	5
Ruminococcaceae_UCG.002	5
Ruminococcaceae_UCG.005	5
sCD30/TNFRSF8	5
sIL.6Rα	5
Acidibacter	4
Chitinophagaceae_unclassified	4
CF: Core fucosylation	4
Faecalibacterium	4
IgG1 H3N4F1: IgG1 glycopeptide with agalactosylated glycan with core fucose	4
IgG1 H5N4F1: IgG1 glycopeptide with digalactosylated glycan with core fucose	4
Isovalerate	4
Lachnospira	4
Prevotella_9	4
Ruminiclostridium_6	4
sCD163	4
Slackia	4
sTNF-R2	4
Variovorax	4
Alphaproteobacteria_unclassified	3
Butyrivibrio	3
HM: High mannose glycans	3
IgG2	3
IgG3	3
IL-2	3
Osteopontin	3
Prevotella_1	3
Psychrobacillus	3
Ruminococcus_2	3
Butyrate	2
Chitinase-3-like 1	2

Citrobacter	2
GCA-900066755	2
IgG4 H3N4F1: IgG4 glycopeptide with agalactosylated glycan with core fucose	2
IL-22	2
Izimaplasmatales_unclassified	2
Lachnospiraceae_UCG.001	2
Muribaculaceae_unclassified	2
Nitrospira	2
Oscillibacter	2
Phascolarctobacterium	2
Ruminococcaceae_NK4A214_group	2
Senegalimassilia	2
TWEAK/TNFSF12	2
Tyzzerella	2
X0319.6G20_unclassified	2
2-methylbutyrate	2
Acidaminococcus	1
Akkermansia	1
Alkanibacter	1
Asteroleplasma	1
Betaproteobacteriales_unclassified	1
Campylobacter	1
Clostridiales_unclassified	1
Coprococcus_2	1
Dielma	1
Erysipelotrichaceae_unclassified	1
Family_XIII_UCG.001	1
FIrmicutes_unclassified	1
Gemella	1
Helicobacter	1
HB: High branching glycans	1
IFN-β	1
IL-11	1
IS-44	1
Legionella	1
Leptotrichia	1
Libanicoccus	1
LIGHT/TNFSF14	1
Microbacterium	1
MMP-1	1
Neisseria	1
Pelomonas	1
Porphyromonas	1
Pseudomonas	1

Raoultella	1
Sediminibacterium	1
S3: Trisialylated.glycans	1

Table S10. Elastic Net selected frequency of all the features for urban v rural group with rankings (in decreasing order) over 100 iterations.

Selected features	Frequency
IFN-γ	53
C-peptide	47
Bifidobacterium	42
Lachnospira	33
Lachnoclostridium	31
Veillonella	29
Sutterella	25
Insulin	24
Chitinase-3-like 1	20
Dialister	20
IL-29/IFN-λ	20
Megamonas	19
CF: Core fucosylation	17
Olsenella	17
IgG2 H3N4F1: IgG2&3 glycopeptides with agalactosylated glycan with core fucose	16
IgG3	16
Prevotella_7	16
G0: Agalactosylated glycans	15
IL-28A/IFN-λ2	15
S0: Neutral (not sialylated) glycans	15
IgG1	14
IgG4 H4N4F1: IgG4 glycopeptide with monogalactosylated glycan with core fucose	14
Intestinibacter	14
Libanicoccus	14
MMP-3	14
Roseburia	14
Senegalimassilia	14
Tyzzerella	14
Age	13
Butyrate	13
Propionate	13
Visfatin	13
FIrmicutes_unclassified	11
S2: Disialylated glycans	10
2-hydroxybutyrate	10
Family_XIII_UCG.001	9
Mitsuokella	9
Muribaculaceae_unclassified	9
AF: Antennary fucosylation	8
Christensenellaceae_R.7_group	8
Comamonas	8
Fusobacterium	8

gp130/sIL-6Rβ	8
IgG2 H4N4F1: IgG2&3 glycopeptides with monogalactosylated glycan with core fucose	8
Prevotella_2	8
Subdoligranulum	8
Butyrivibrio	7
IgA	7
Lachnospiraceae_ND3007_group	7
2-methylbutyrate	7
Bacteroides	6
B: Bisection (Glycans with bisecting GlcNAc)	6
IgG4	6
Lachnospiraceae_UCG.001	6
Leptin	6
Osteocalcin	6
Erysipelotrichaceae_unclassified	5
IgG4 H5N4F1S1: IgG4 glycopeptide with digalactosylated and monosialylated glycan with core fucose	5
MMP-1	5
Romboutsia	5
Ruminococcaceae_UCG.013	5
Solobacterium	5
CAG-56	4
Citrobacter	4
Clostridium_sensu_stricto_1	4
Isovalerate	4
Klebsiella	4
PAI-1	4
Ruminococcus_1	4
vadinBE97_unclassified	4
Anaeroplasma	3
Erysipelotrichaceae_UCG.003	3
GIP	3
GLP-1	3
Haemophilus	3
HM: High mannose glycans	3
IgG1 H4N4F1: IgG1 glycopeptide with monogalactosylated glycan with core fucose	3
IL-27 (p28)	3
Ruminococcaceae_NK4A214_group	3
Slackia	3
Victivallis	3
Acinetobacter	2
Aeromonas	2
Alloprevotella	2

Asinibacterium	2
Asteroleplasma	2
Barnesiella	2
Coprococcus_2	2
Dorea	2
Elusimicrobium	2
Enterococcus	2
Flavonifractor	2
Gastranaerophilales_unclassified	2
Ghrelin	2
Glucagon	2
Granulicatella	2
IgG2	2
IgM	2
Lachnospiraceae_UCG.004	2
LIGHT/TNFSF14	2
Paraprevotella	2
Pentraxin 3	2
Alistipes	1
Anaerostipes	1
APRIL/TNFSF13	1
Bacteroidales_unclassified	1
Bilophila	1
Caproate	1
Chitinophagaceae_unclassified	1
G2: Digalactosylated glycans	1
Erysipelatoclostridium	1
Escherichia/Shigella	1
HB: High branching glycans	1
IgG2 H5N4F1: IgG2&3 glycopeptides with digalactosylated glycan with core fucose	1
IgG4 H3N4F1: IgG4 glycopeptide with agalactosylated glycan with core fucose	1
IL-11	1
IL-22	1
Lachnospiraceae_unclassified	1
LB: Low branching glycans	1
MMP-2	1
G1: Monogalactosylated glycans	1
Oxalobacter	1
Phascolarctobacterium	1
Resistin	1
Ruminococcaceae_UCG.005	1
sCD30/TNFRSF8	1
sIL.6Rα	1

G4: Tetragalactosylated glycans	1
S4: Tetrasialylated glycans	1
Treponema_2	1
G3: Trigalactosylated glycans	1
S3: Trisialylated glycans	1
TSLP	1
UBA1819	1
Valerate	1

Table S11. Elastic Net selected frequency of all the features for normal vs overweight groups with rankings in decreasing order) over 100 iterations.

Selected features	Frequency
Prevotellaceae_NK3B31_group	67
Dialister	64
C-peptide	49
Gastranaerophilales_unclassified	49
Glucagon	49
CAG-56	48
Sutterella	48
Haemophilus	47
Muribaculaceae_unclassified	47
AF: Antennary fucosylation	45
Treponema_2	43
Prevotella_2	41
Age	38
Acinetobacter	37
Aeromonas	37
Granulicatella	37
Paraprevotella	36
Ghrelin	34
Solobacterium	34
Enterococcus	32
Ruminococcaceae_UCG.013	32
Bilophila	31
UBA1819	31
Erysipelotrichaceae_UCG.003	30
Asteroleplasma	29
Romboutsia	29
APRIL/TNFSF13	27
MMP-2	27
Anaeroplasma	26
Dorea	26
Insulin	26
Anaerostipes	25
IgG1 H5N4F1S1: IgG1 glycopeptide with digalactosylated and monosialylated glycan with core fucose	24
Lachnospiraceae_UCG.001	24
vadinBE97_unclassified	24
Veillonella	23
S2: Disialylated glycans	22
Pentraxin 3	22
2-hydroxybutyrate	22
Mollicutes_RF39_unclassified	20
TWEAK/TNFSF12	20
Leptin	19
PAI-1	19

Bifidobacterium	18
Butyricicoccus	18
Erysipelotrichaceae_unclassified	18
IgG3	18
Subdoligranulum	18
Succinivibrio	18
Erysipelotrichaceae_UCG.004	16
Parasutterella	16
Acidaminococcus	15
Barnesiella	15
Mitsuokella	15
MMP-1	15
Akkermansia	14
IgG4	14
Lachnospira	14
Blautia	13
IgM	12
MMP-3	12
Olsenella	12
Anaerovibrio	11
Comamonas	11
IgG4 H5N4F1: IgG4 glycopeptide with digalactosylated glycan with core fucose	11
IL-11	11
Lachnospiraceae_AC2044_group	11
Osteopontin	11
Clostridium_sensu_stricto_1	10
G2: Digalactosylated glycans	10
Enterobacteriaceae_unclassified	10
Fournierella	10
GLP-1	10
Osteocalcin	10
Escherichia/Shigella	9
Flavonifractor	9
IgG4 H4N4F1: IgG4 glycopeptide with monogalactosylated glycan with core fucose	9
Lachnoclostridium	9
Betaproteobacteriales_unclassified	8
Butyrate	8
Butyrivibrio	8
Campylobacter	8
Fusobacterium	8
Gemella	8
Leptotrichia	8
Neisseria	8
Porphyromonas	8

Prevotellaceae_unclassified	8
Pseudocitrobacter	8
Ralstonia	8
Asinibacterium	7
Bacteroidales_unclassified	7
gp130/sIL.6Rb	7
IgA	7
IgG1	7
Klebsiella	7
Lachnospiraceae_UCG.004	7
Megamonas	7
G1: Monogalactosylated glycans	7
Oribacterium	7
Prevotella_9	7
Rhodospirillales_unclassified	7
Visfatin	7
2-methylbutyrate	7
Alistipes	6
Caproate	6
Clostridiales_unclassified	6
Erysipelatoclostridium	6
GIP	6
IgG1 H5N4F1: IgG1 glycopeptide with digalactosylated glycan with core fucose	6
IgG2	6
IgG4 H3N4F1: IgG4 glycopeptide with agalactosylated glycan with core fucose	6
Lactobacillus	6
Prevotella_7	6
Resistin	6
Roseburia	6
sCD30/TNFRSF8	6
G4: Tetragalactosylated glycans	6
S4: Tetrasialylated.glycans	6
Agathobacter	5
Chitinase-3-like 1	5
Christensenellaceae_R.7_group	5
Coprococcus_2	5
Coprococcus_3	5
IFN-γ	5
S0: Neutral (not sialylated) glycans	5
sTNF-R2	5
Catenibacterium	4
Collinsella	4
Coprococcus_1	4
Lachnospiraceae_NK4A136_group	4
Parabacteroides	4

Phascolarctobacterium	4
Rikenellaceae_RC9_gut_group	4
Ruminiclostridium_6	4
Ruminococcaceae_UCG.010	4
Ruminococcus_1	4
Ruminococcus_2	4
sCD163	4
Streptococcus	4
IgG2 H3N4F1: IgG2&3 glycopeptides with agalactosylated glycan with core fucose	3
IgG2 H5N4F1S1: IgG2&3 glycopeptides with digalactosylated and monosialylated glycan with core fucose	3
IL-28A/IFN-λ2	3
IL-29/IFN-λ1	3
IL-8	3
Lachnospiraceae_unclassified	3
Propionate	3
Ruminococcaceae_NK4A214_group	3
sIL-6Rα	3
TSLP	3
Valerate	3
Alloprevotella	2
B: Bisection (Glycans with bisecting GlcNAc)	2
HM: High mannose glycans	2
Holdemanella	2
IFN-α2	2
IFN-β	2
IgG1 H3N4F1: IgG1 glycopeptide with agalactosylated glycan with core fucose	2
IgG1 H4N4F1: IgG1 glycopeptide with monogalactosylated glycan with core fucose	2
IgG2 H4N4F1: IgG2&3 glycopeptide with monogalactosylated glycan with core fucose	2
IgG2 H5N4F1: IgG2&3 glycopeptide with digalactosylated glycan with core fucose	2
IL-20	2
IL-22	2
IL-27 (p28)	2
Odoribacter	2
Ruminococcaceae_UCG.014	2
G3: Trigalactosylated glycans	2
S3: Trisialylated glycans	2
G0: Agalactosylated glycans	1
CF: Core fucosylation	1
Elusimicrobium	1
Faecalibacterium	1
Family_XIII_AD3011_group	1

HB: High branching glycans	1
IgG4 H5N4F1S1: IgG4 glycopeptide with digalactosylated and monosialylated glycan with core fucose	1
IL-32	1
Isobutyrate	1
Isovalerate	1
Izimaplasmatales_unclassified	1
LIGHT/TNFSF14	1
Oxalobacter	1
Ruminococcaceae_UCG.002	1
Ruminococcaceae_UCG.003	1
Slackia	1
sTNF-R1	1

Table S12. Elastic Net selected frequency of all the features for normal vs pre-obese groups with rankings (in decreasing order) over 100 iterations.

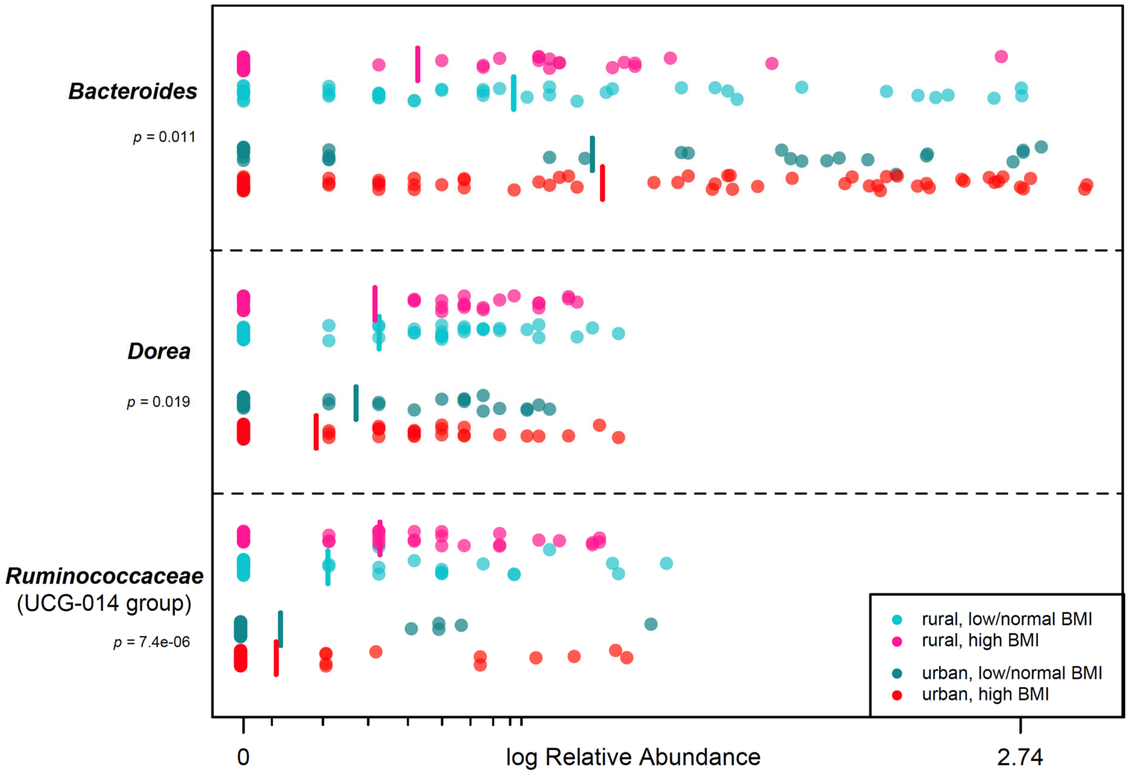

Figure S1. Log-transformed relative abundance of significantly differential genera between participants with high (≥23) or low/normal (<23) BMI score in rural and/or urban groups, as determined by Linear discriminant analysis effect size (LEfSe). *Ruminoccaceae* (group UCG-014) was significantly different in comparisons using both all samples and within urban samples alone.

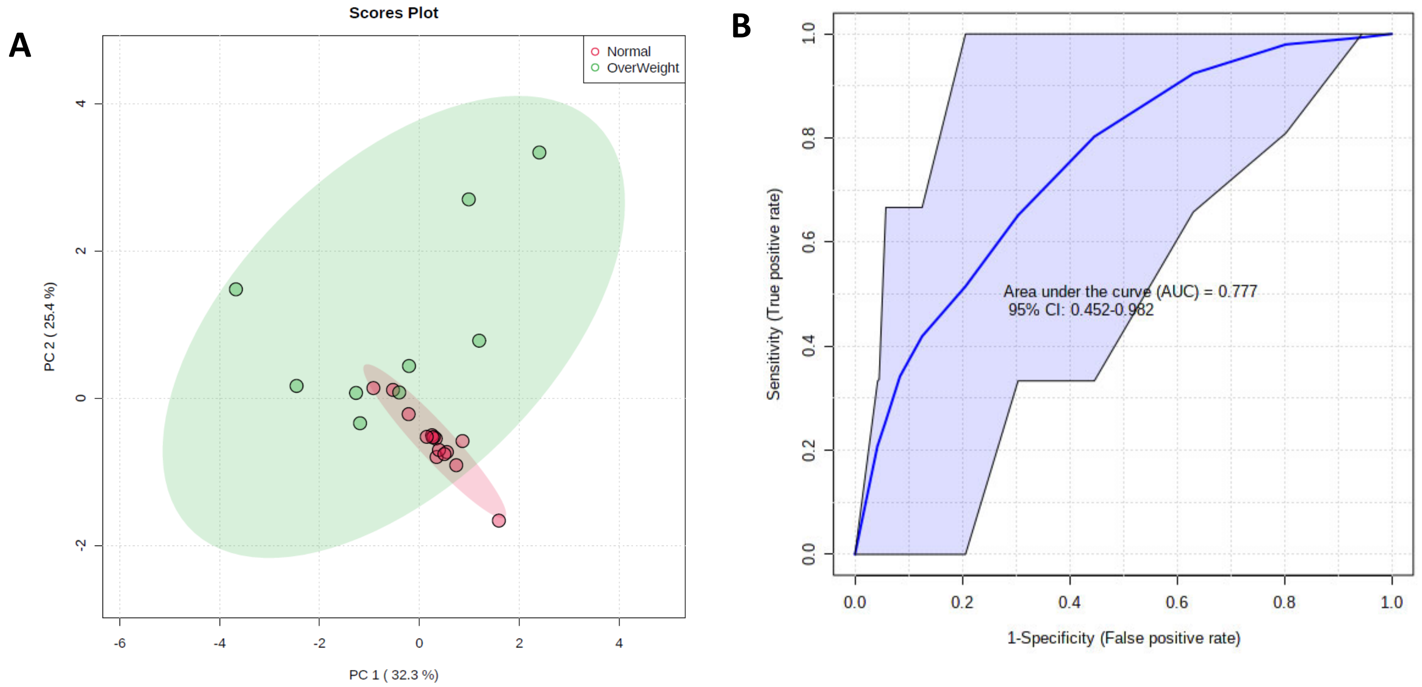

Figure S2. A) Principal component analysis (PCA) score plot performed on the Overweight vs. Normal population demonstrating clustering of subjects within samples. BMI ranges were pre-defined using WHO Asian BMI classifications: underweight normal (18.5-22.9); overweight (23-

24.9) categories. PCA analysis used five selected discriminatory features (IFN-gamma, C-peptide, *Lachnospira, Bifidobacterium, Lachnoclostridium*) between Normal vs. Overweight using Elastic net analysis. Overweight samples (in green) are more dispersed compared to the normal samples (in red). B) Area under the curve (AUC) is shown using the logistic regression method and five discriminatory features from the elastic net method. A corresponding confidence interval is also calculated as a shaded area.

A

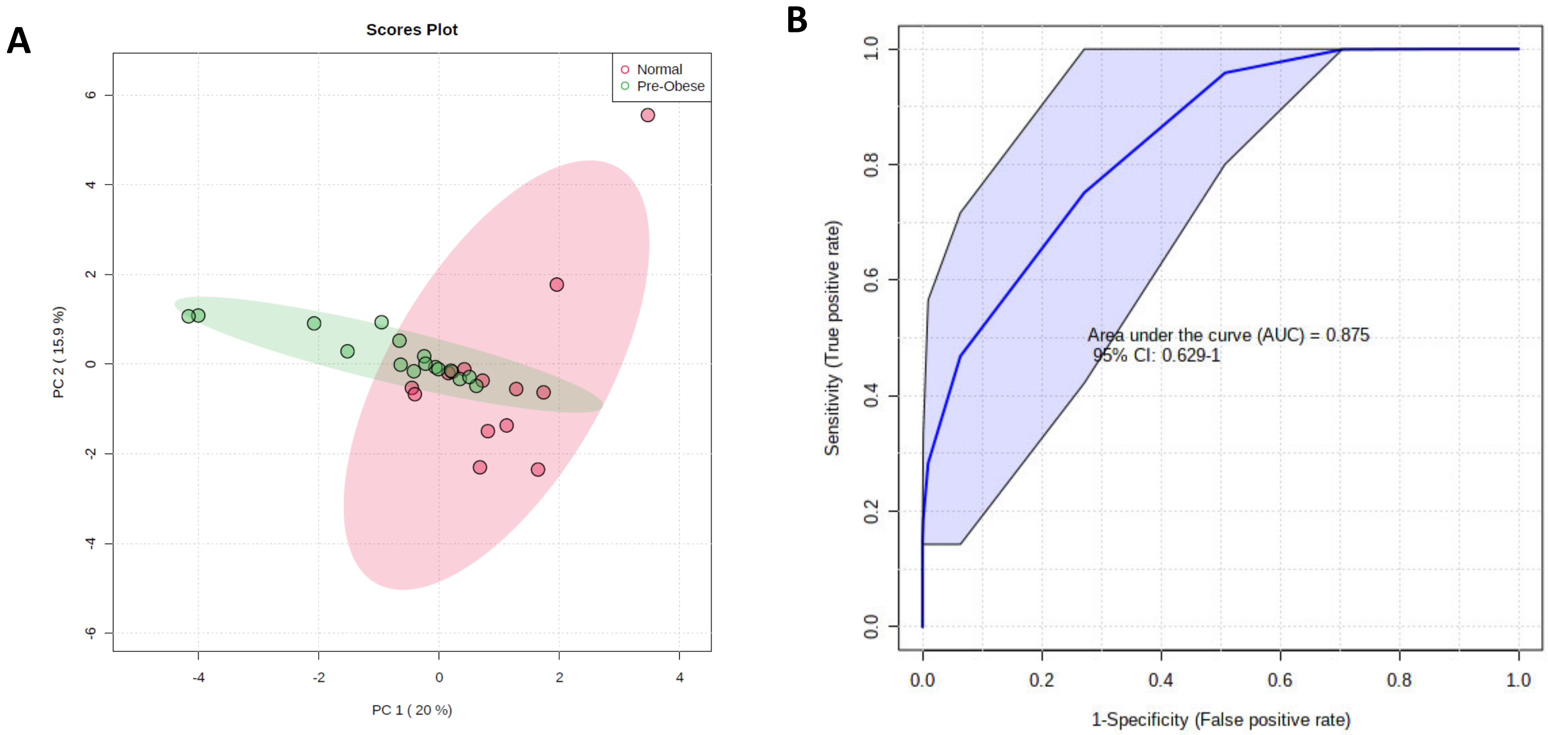

Figure S3. A) Principal component analysis (PCA) score plot performed on the Pre-Obese vs. Normal population demonstrating clustering of subjects within samples. BMI ranges were pre-defined using WHO Asian BMI classifications: normal (18.5-22.9); pre-obese (25-29.9)

categories. PCA analysis used twelve selected discriminatory features (*Prevotellaceae_NK3B31_group, Dialister*, Glucagon, C-peptide, *Prevotella_2, Antennary fucosylation (AF), CAG-56, Muribaculaceae_unclassified, Haemophilus, Sutterella, Treponema_2, Gastranaerophilales_unclassified*) between Normal vs. Pre-Obese using Elastic net analysis. Pre-Obese (in green) and normal BMI samples (in red) seem to be separating from each other. B) Area under the curve (AUC) is shown using the logistic regression method and twelve discriminatory features from the elastic net method. A corresponding confidence interval is also calculated as a shaded area.
